# Fabrication of Aligned Polycaprolactone Nanofibrillar Bundles to Control Cell‐Mediated Collagen Deposition in Engineered Cartilage

**DOI:** 10.1002/adma.75138

**Published:** 2026-09-24

**Authors:** Alba Pueyo Moliner, Lennard Spauwen, James L. Martin Robinson, Ángela García Grech, Isabelle Martinier, Sara Grasselli, Aylin Kara Özenler, Matthias Ryma, Jürgen Groll, Mylène de Ruijter, Jos Malda

**Affiliations:** ^1^ University Medical Center Utrecht & Regenerative Medicine Center Utrecht Utrecht the Netherlands; ^2^ Department of Equine Sciences Faculty of Veterinary Sciences Utrecht University Utrecht the Netherlands; ^3^ University of Applied Sciences Utrecht Utrecht the Netherlands; ^4^ Department for Functional Materials in Medicine and Dentistry Institute for Functional Materials and Biofabrication and Center of Polymers for Life (CPL) University of Würzburg Würzburg Germany

**Keywords:** articular cartilage, collagen, melt electrofibrillation, melt electrowriting, nanofibrillar bundles

## Abstract

The structural anisotropy of collagen is essential for the function of tissues such as articular cartilage, yet current biofabrication strategies fail to reproduce this hierarchical organization. Melt electrowriting (MEW) is widely used to fabricate mechanically stable polycaprolactone (PCL) scaffolds for reinforcing hydrogels in cartilage tissue engineering, but these constructs typically develop randomly organized collagen networks during chondrogenic differentiation, both in vitro and in vivo. Here, we introduce melt electrofibrillation (MEF), a variation of MEW, as a promising strategy to produce nanofibrillar PCL scaffolds with defined architectures for cartilage tissue engineering. We demonstrate that MEF PCL scaffolds promote enhanced cellular alignment and retention while consistently guiding the organization of a collagen type II‐rich extracellular matrix. As a result, MEF provides a tunable, printable scaffold for directing organized cartilage matrix formation. This approach represents a significant step toward engineering biomimetic cartilage and potentially other aligned, collagen‐rich tissues with controlled structural organization.

## Graphical abstract

Melt electrofibrillation (MEF) enables the fabrication of nanofibrillar PCL scaffolds with defined architectures that guide cellular alignment and retention during cartilage tissue engineering. Unlike conventional melt electrowriting, MEF consistently directs collagen type II‐rich extracellular matrix organization, providing a tunable platform to reproduce the structural anisotropy of native cartilage and engineer biomimetic tissues.

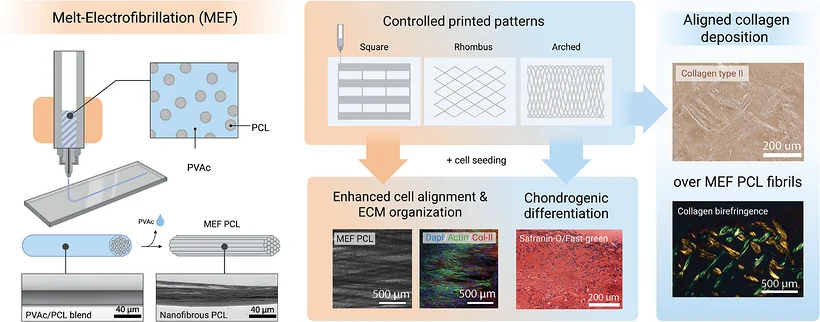

## 1 Introduction

Collagen is the most abundant protein in mammals and serves as the primary structural component of the extracellular matrix (ECM) [1]. Among the various collagen types, collagen type II is the predominant fibrillar collagen in cartilaginous tissues and the corpus vitreum of the eye [2, 3]. Collagen type II accounts for approximately 60% of the dry weight of articular cartilage. It is organized into fibrils with diameters ranging from ∼30 to 200 nm, which further assemble into larger fiber bundles [4]. In cartilage, these fibrils exhibit a hierarchical, depth‐dependent, arch‐like organization: aligned perpendicular to the surface in the deep zone, randomly oriented in the middle zone, and parallel to the articular surface in the superficial zone. This architecture constrains the swelling pressure generated by hydrated proteoglycans and enables cartilage to withstand cyclic compressive loading throughout a lifetime [5, 6, 7].

Despite these remarkable properties, articular cartilage has a limited intrinsic regenerative capacity [8]. Following injury, cartilage damage typically results in the formation of scar tissue with unorganized collagen, which is mechanically inferior [6]. If left untreated, focal cartilage defects are a major contributor to osteoarthritis (OA), a degenerative joint disease affecting millions worldwide [9]. Current therapeutic approaches include osteochondral grafts, injectable hydrogels (such as synthetic hydrogels, e.g., PEG formulations, or ECM‑derived, e.g., hyaluronic acid‐ or chitosan‐based hydrogels), and cell‐based strategies (e.g., autologous chondrocyte implantation) [1, 10, 11]. Yet, their long‐term success is limited by the assumption that the collagen network in mature tissue retains the capacity to remodel [8, 12].

This collagen network is established during late fetal and early postnatal development, and its metabolic turnover in adulthood is negligible [13, 14, 15, 16, 17, 18, 19, 20], providing a compelling explanation for the limited long‐term success of current regenerative strategies [8, 21].

Biofabrication strategies have emerged as a promising tool for the development of novel cartilage implants. For example, melt electrowriting (MEW) has been used to fabricate reinforced polycaprolactone (PCL) scaffolds that mimic the mechanical function of native collagen [22, 23, 24, 25]. These load‐bearing (osteo)chondral constructs are intended as a plug‐like implant for the treatment of cartilage defects [26, 27, 28]. However, the eventual in vivo degradation of PCL fibers emphasizes the need for strategies that not only provide initial reinforcement but also promote the formation of a native‐like collagen type II architecture [8].

While the alignment of collagen type I networks has been described [29, 30], efforts to control collagen type II organization have so far failed to replicate the native depth‐dependent architecture [5, 27, 31, 32, 33]. The most recent approaches guide differentiating cells or cell aggregates through physical confinement within voids formed by printed scaffolds or oriented hydrogel channels [31, 32, 34]. However, such strategies rely on passive confinement, meaning cells are left to deposit their matrix within a predefined void, with limited means to dictate the orientation and hierarchy of the collagen that is formed [5]. This leaves a critical gap: no current method can directly control the architecture of cell‐deposited collagen type II.

In our research, we aim to close this gap with a more direct way to guide collagen organization in cartilage implants. To this end, we used melt electrofibrillation (MEF), a fabrication strategy that generates aligned bundles of PCL nanofibrils with diameters down to ∼300 nm—an order of magnitude below conventional MEW fibers (∼10 µm) [35]. Unlike other nanofiber printing techniques, MEF retains the ability to “write” fibrils in predefined patterns, offering precise control over fiber orientation and scaffold anisotropy. We compared conventional MEW scaffolds with MEF fibrillar scaffolds, evaluating printability, mechanical performance, and the scaffold effects on cell alignment, distribution, and collagen type II organization during in vitro chondrogenesis.

Since the mature collagen network cannot be remodeled, we reasoned that guiding matrix organization at the stage it is being formed, rather than relying on post‐hoc remodeling, offers the most viable route to replicate native‐like tissue. We therefore hypothesized that MEF‐generated nanofibrillar bundles provide topographical cues that better direct the organization of newly deposited collagen type II. By linking scaffold microarchitecture directly to matrix assembly, this work positions MEF as a strategy that moves toward the fabrication of functionally organized cartilage implants, with potential relevance for other collagen‐rich tissues.

## 2 Results and Discussion

### 2.1 MEF

To fabricate PCL nanofibrillar bundles, MEW was adapted by printing a blend of two immiscible polymers, rather than neat PCL (Figure 1A), thereby inducing flow‐driven separation during the printing process [35]. Specifically, a blend of 30% PCL and 70% polyvinyl acetate (PVAc), hereafter referred to as pre‐MEF PVAc/PCL, was successfully printed using an applied voltage of 3–6 kV, a constant air pressure of 0.5 bar, and collector speeds ranging from 2 to 10 mm/s. Under these conditions, continuous microscale fibers with diameters between 24 ± 5 and 40 ± 7 µm were obtained (Figure 1B). This polymer ratio was selected based on previous literature, which demonstrated that a 30% PCL/70% PVAc blend (*w/w*) provided an optimal compromise between internal fiber morphology and sufficient mechanical stability for printing [35, 36].

**FIGURE 1:**
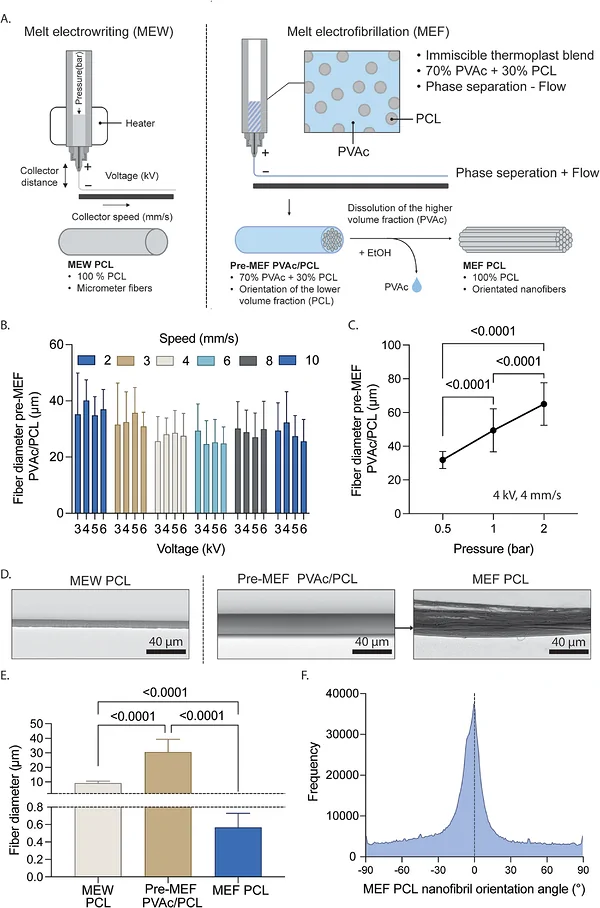
(A) Schematic overview of the fabrication process and material composition, illustrating MEW printing of pure PCL (MEW PCL) and printing of the pre‐MEF PCL/PVAc polymer blend, including PVAc dissolution/fibrillation to obtain nanofibrillar MEF PCL bundles of fibrils. (B) Influence of applied voltage (3, 4, 5, and 6 kV) and collector speed (2, 3, 4, 6, 8, 10 mm/s) on fiber diameter during MEW printing (*n* = 27). (C) Relationship between applied printing pressure (0.5, 1, 2 bar) and resulting fiber diameter (µm) for the pre‐MEF PCL/PVAc polymer blend (*n* = 27). (D) Representative scanning electron microscopy (SEM) images of MEW PCL individual fiber, pre‐MEF PVAc/PCL polymer blend fiber, and MEF PCL‐derived bundle of nanofibrils. (E) Quantitative comparison of fiber diameters (µm) for MEW PCL, pre‐MEF PVAc/PCL blend fibers, and individual MEF PCL nanofibrils (*n* = 20). (F) Orientation angle (°) distribution of MEF PCL nanofibrils after the fibrillation process, corresponding to the representative image shown in 1D.

Pre‐MEF PVAc/PCL fiber diameter was largely unaffected by variations in applied voltage; increasing the collector speed resulted in reduced fiber diameter; and increasing the applied pressure resulted in a significant increase in fiber diameter (Figure 1C), consistent with previous observations for MEW PCL [37]. The nozzle‐to‐collector distance was fixed at 3 mm, which provided the most stable print quality in our setup, falling within the low‐millimeter range previously reported for MEF fabrication [35, 36]. Notably, the diameters of pre‐MEF PVAc/PCL polymer blend fibers were approximately 3.5‐fold larger than those produced by conventional MEW PCL (9 ± 1 µm; Figure 1D,E). This increase is likely due to differences in the rheological and electrohydrodynamic properties of the polymer blend, such as higher viscosity during jetting compared to MEW PCL [38].

Selective dissolution of PVAc using 70% ethanol revealed the highly aligned bundles of PCL nanofibrils (Figure 1D), hereafter referred to as MEF PCL. This process, termed *fibrillation*, exposes the nanofibrillar structure of the lower‐volume immiscible polymer, in this case PCL. Fibrillation occurs during printing as a result of flow‐induced phase separation, making the selection of a higher‐volume thermoplastic component (here PVAc) that can be selectively removed after printing a critical step (Figure 1A) [35]. Following dissolution, the average diameter of individual MEF PCL fibrils was 560 ± 160 nm, while the overall bundle diameter expanded (30 ± 9 µm) but remained comparable to that prior to washing (Figure 1E). These findings are consistent with previous reports showing that MEF enables the fabrication of highly aligned PCL nanofibrillar bundles (Figure 1F) with continuous internal fibrillar architecture, in which nanofibrils are distributed throughout the entire bundle rather than being confined to the surface (Figure 1D) [35, 39]. Compared with conventional electrospinning, MEF offers several advantages for generating organized fibrillar architectures. Electrospinning is a well‐established technique for producing nanofibers, but it typically yields randomly deposited, two‐dimensional fiber mats with limited control over fiber placement and overall architecture, and directional alignment generally requires auxiliary strategies such as rotating collectors or patterned electrodes [40, 41]. MEF, in contrast, retains the direct‐write precision of MEW, enabling fibrils to be deposited in predefined patterns and three‐dimensional architectures, while achieving fibril diameters in the nanometer range. This combination of controlled deposition and sub‐micron fibril dimensions, together with the capacity to generate organized three‐dimensional architectures, is not readily achieved by electrospinning alone. Nevertheless, precise control over the nanofibril bundle diameter following PVAc dissolution remains limited.

Lastly, FT‐IR spectra supported substantial removal of PVAc from the MEF PCL nanofibrils (Figure S1), consistent with previous Raman spectroscopy findings [42]. Mass‐loss analysis further confirmed removal of the sacrificial PVAc phase, with fibrillated scaffolds exhibiting around 70.68% mass loss, consistent with the theoretical PVAc content of a 30% PCL/70% PVAc blend (*w/w*) (Figure S1). All subsequent fabrication, mechanical, and biological analyses were therefore performed on MEF PCL nanofibrils from which PVAc had been substantially removed.

### 2.2 Printability and Printing Accuracy

Next, we extended the printability of pre‐MEF PVAc/PCL to fabricate larger scaffolds with more complex architectures. This enabled assessment of the ability of nanofibrillar PCL to conform to predefined geometries. Structured pre‐MEF PVAc/PCL polymer blend scaffolds were compared to neat PCL MEW constructs with distinct geometries, including box and rhombus patterns [39].

Box‐shaped scaffolds (Figure 2A), commonly used as hydrogel reinforcement in osteochondral implants [25], were fabricated at three thickness levels (10, 25, and 50 layers) to assess how fibril density and stacking influence the topographical definition of the constructs. Profilometric analysis revealed distinct height differences between fiber crossover points (“high” regions, H) and the intermediate zones (“low” regions, L). For 10 layers, heights measured 420 ± 130 µm (H) and 185 ± 66 µm (L); for 25 layers, 974 ± 96 µm (H) and 553 ± 85 µm (L); and for 50 layers, 1080 ± 230 µm (H) and 510 ± 120 µm (L) (Figure 2B,C). In pre‐MEF PVAc/PCL scaffolds, the overall height plateaued beyond 25 layers, indicating limited stacking efficiency with increasing deposition. In contrast, neat MEW PCL scaffolds exhibited a consistent increase in height with each additional layer. This discrepancy likely reflects reduced interlayer cohesion and electrostatic fiber repulsion in the polymer blend, leading to greater lateral displacement during printing. This effect is pronounced at fiber crossover points, where reduced stackability results in a lower scaffold height.

**FIGURE 2:**
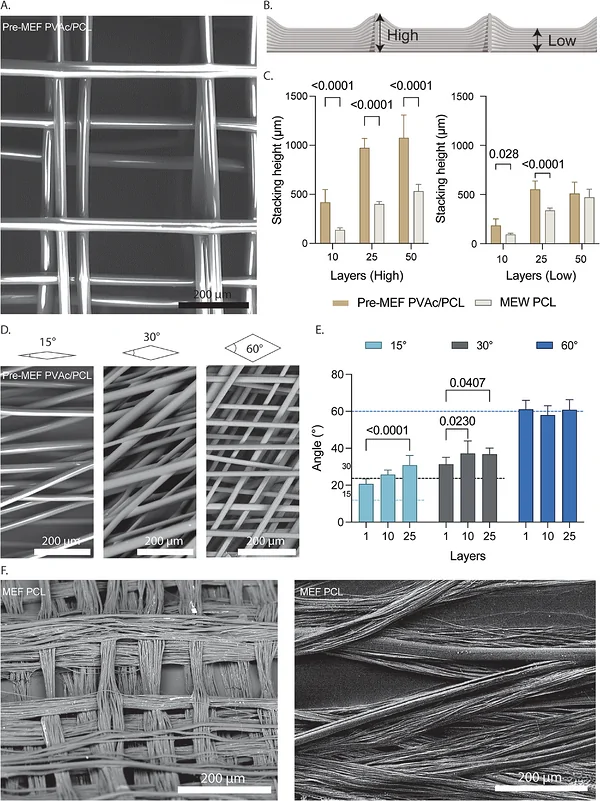
Evaluation of printing accuracy and structural fidelity of scaffolds fabricated from MEW PCL and pre‐MEF PVAc/PCL polymer blend. (A) Representative SEM image of box‐shaped pre‐MEF PVAc/PCL scaffold (5 layers). (B) 3D surface render of box‐shaped scaffold topography, highlighting height differences at fiber crossover points (“high” regions) and intermediate areas (“low” regions). (C) Quantitative analysis of stacking height in high (left) and low (right) regions for MEW PCL and pre‐MEF PVAc/PCL blend scaffolds printed at 10, 25, and 50 layers (*n* = 9, 3 samples, 3 measurements per print). (D) SEM images of rhombus‐shaped pre‐MEF PVAc/PCL scaffolds with designed fiber angles of 15°, 30°, and 60° (25 layers). (E) Quantitative analysis of printing accuracy for rhombus‐shaped pre‐MEF PVAc/PCL architectures with target angles of 15°, 30°, and 60°, printed at 1, 10, and 25 layers (*n* = 9, 3 samples, 3 measurements per print). (F) Representative images of MEF PCL nanofibrillar bundles after selective dissolution of the sacrificial PVAc phase, shown in box and rhombus geometries (5 layers).

Pre‐MEF PVAc/PCL scaffolds exhibited a greater height difference between high and low regions compared to MEW PCL scaffolds, likely due to the larger average fiber diameter in the polymer blend constructs (30 ± 9 µm vs. 9 ± 1 µm for neat PCL; Figure 1E). The increased fiber diameter also limits the minimum achievable fiber spacing, as thicker fibers are more prone to bridging across narrow gaps, thereby reducing stacking fidelity and printability [43]. Moreover, insufficient spacing between adjacent fibers further impairs proper layer‐by‐layer deposition, compromising overall architectural precision.

In addition to box‐shaped scaffolds, rhombus‐shaped pre‐MEF PVAc/PCL scaffolds with angles of 15°, 30°, and 60° were fabricated at 1, 10, and 25 layers to further evaluate the influence of fiber orientation and architecture on printing accuracy (Figure 2D). Constructs with lower rhombus angles proved more challenging to print with angular fidelity. For 15° constructs, the printed angles progressively deviated from intended geometry with increasing layer number: 21° ± 3° at 1 layer, 26° ± 2° at 10 layers, and 31° ± 5° at 25 layers (Figure 2E). In contrast, constructs with 30° and 60° angles maintained greater dimensional accuracy at 25 layers, with measured angles of 39° ± 3° and 61° ± 5°, respectively. The increasing deviation observed at 15° likely results from reduced stackability, leading to lateral fiber displacement and decreased deposition precision. Additionally, smaller rhombus angles impose geometric constraints by limiting the spacing between intersecting fibers, which, combined with larger fiber diameter, further reduces printing accuracy [43]. Conversely, larger rhombus angles, approaching a box‐like configuration (∼90°), provide greater jet stability and improved overall printability [43]. Such limitations are not observed for rhombus‐shaped MEW PCL scaffolds (Figure S2) [44].

These findings indicate that the printability of pre‐MEF PVAc/PCL is dependent not only on the selected architecture but also on the interaction between geometry and deposition conditions. In particular, the deviation observed for low‐angle rhombi is likely related to the limited stackability of the polymer blend and the geometric constraints imposed by the narrow intersections. Despite optimization of the printing parameters, these effects remained evident, suggesting that the observed deviation represents a limitation of the current pre‐MEF formulation and printing process rather than simply a lack of parameter optimization. Future process development could therefore focus on geometry‐specific strategies, such as compensating the printing path for predictable lateral fiber displacement or adapting the deposition sequence in regions with sharp angles or closely spaced intersections [45]. Such approaches may help improve geometric fidelity when translating pre‐MEF to more complex three‐dimensional architectures.

After dissolving the sacrificial PVAc component of the pre‐MEF PVAc/PCL, scaffolds retained their overall morphology (Figure 2F). Still, removal of PVAc substantially reduced inter‐fiber cohesion, compromising mechanical integrity and resulting in increased brittleness. This reflects a trade‐off in the MEF process, where high PVAc content is necessary for nanoscale fibril formation via phase separation, but results in increased interfibrillar void space and suboptimal stacking after its removal. This also highlights that the mechanical properties of the final scaffold cannot be optimized independently of the pre‐MEF printing process.

Several strategies could therefore be explored to improve post‐leaching integrity. First, the PVAc/PCL ratio could be optimized to identify the lowest PVAc content that still produces the desired nanofibrillar morphology, thereby reducing the void volume generated during leaching. Second, printing parameters could be adjusted to increase the contact and overlap between successive PCL‐containing fibers before PVAc removal, potentially improving the continuity of the remaining PCL network. In addition, combining the pre‐MEF component with a reinforcing framework of neat PCL MEW fibers could provide mechanical reinforcement while retaining the nanofibrillar surface generated by MEF. Such a hybrid strategy may be particularly advantageous for larger or load‐bearing constructs, where the nanofibrillar component primarily provides topographical guidance cues, while the MEW component provides structural reinforcement.

Collectively, these results demonstrate that MEF enables the fabrication of architectures with controlled nanoscale topographies and defined fiber organization. Compared with conventional electrospinning, MEF provides greater control over fiber placement and scaffold geometry, resulting in improved architectural reproducibility and enabling the fabrication of predefined three‐dimensional constructs while retaining a nanofibrillar morphology. However, the observed limitations, including reduced stackability, loss of angular fidelity in highly constrained geometries, and reduced mechanical integrity following PVAc removal, highlight the need for coordinated optimization of blend composition and printing path design.

### 2.3 Mechanical Properties

Native cartilage relies strongly on fluid pressurization for load distribution and energy dissipation under compression. In tissue engineering, MEW PCL scaffolds are used to reinforce soft hydrogels, where structural support arises from hydrogel confinement and stabilization of the fiber network at intersection points [25, 46, 47, 48]. Covalently cross‐linked hydrogels such as GelMA can further tension the MEW network, contributing to load transfer and mechanical support within the composite [51]. These reinforced hydrogel‐fiber constructs have therefore been explored as chondral implants for cartilage defect repair, where the combination of a hydrated hydrogel matrix and reinforcing MEW scaffold can provide both a cell‐supportive environment and mechanical stability similar to native cartilage [49, 50].

Given the more open nanofibrillar architecture of MEF PCL, we aimed to investigate its mechanical reinforcing capacity relative to MEW. Therefore, the mechanical performance of MEW and MEF PCL scaffolds was evaluated under tensile and compressive loading, including static and cyclic compression and time‐dependent stress relaxation behavior (Figure 3 and Figure S3). Under uniaxial tensile testing, MEF PCL nanofibrillar scaffolds exhibited a significantly lower ultimate tensile modulus (2.3 ± 2.2 MPa) compared to MEW scaffolds (7.0 ± 2.0 MPa), corresponding to an approximate 67% reduction (Figure 3A,B). This decrease is consistent with the heterogeneous, discontinuous architecture of MEF scaffolds, which fail more variably than the fused MEW network.

**FIGURE 3:**
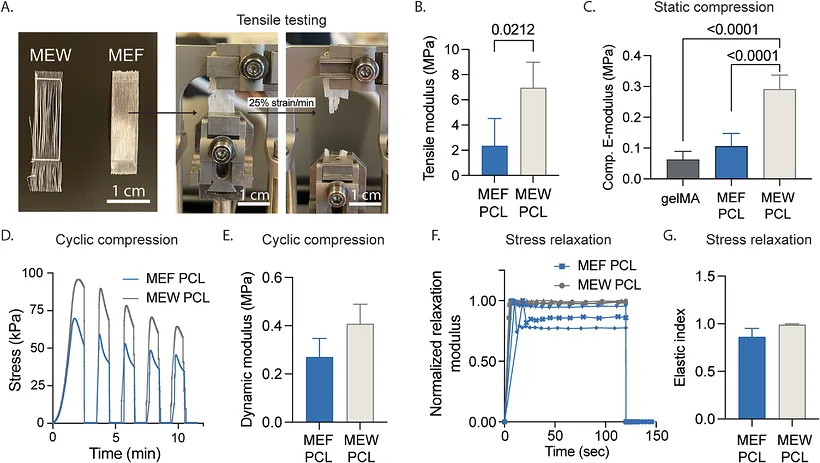
Mechanical characteristics of PCL scaffolds fabricated via MEF PCL and MEW PCL. (A) Experimental setup for tensile testing, including a stabilizing frame to prevent premature damage during sample mounting. (B) Comparison of tensile modulus between MEF PCL and MEW PCL fibers (*n* = 4). (C) Compressive modulus of GelMA constructs, either unreinforced or reinforced with MEF PCL and MEW PCL scaffolds (*n* = 5). (D) Compressive stress as a function of time during cyclic loading between 10% and 30% strain for MEW‐ and MEF PCL‐reinforced constructs (*n* = 3). (E) Dynamic modulus over five consecutive loading cycles (*n* = 3). (F) Normalized relaxation modulus as a function of time during stress‐relaxation testing (*n* = 3). (G) Elastic index of MEW‐ and MEF‐reinforced constructs at the end of the stress‐relaxation period (elastic index, defined as the final relaxation modulus normalized to the maximum modulus of each sample) (*n* = 3).

To assess their reinforcing capacity under static compression, both scaffold types were embedded in gelMA, and the compressive modulus (E‐modulus) of the resulting constructs was measured. Non‐reinforced gelMA hydrogels exhibited the lowest modulus (62 ± 27 kPa), whereas MEW PCL‐reinforced gelMA showed a significantly higher modulus (291 ± 45 kPa), consistent with previous reports (Figure 3C) [25, 51]. In contrast, MEF PCL‐reinforced gelMA constructs displayed only a modest increase (106 ± 41 kPa) compared to gelMA alone, remaining substantially lower than MEW PCL‐reinforced scaffolds. This limited mechanical improvement is likely due to the highly open nanofibrillar network formed after PVAc dissolution, which lacks inter‐fiber fusion and cohesion at crossover points, thereby reducing hydrogel confinement and resistance to compressive deformation. Both MEW and MEF PCL scaffolds showed a gradual decrease in peak stress with repeated loading. However, MEW displayed a higher dynamic modulus (0.41 ± 0.08 MPa) compared with MEF (0.27 ± 0.08 MPa) (Figure 3D,E). Stress‐relaxation testing further showed that MEW‐reinforced constructs retained a slightly greater proportion of their initial modulus than MEF‐reinforced constructs, indicating a modestly lower degree of stress relaxation under sustained deformation (Figure 3F,G).

Overall, MEW provided greater mechanical reinforcement than MEF across the tested loading conditions. Although MEF contributed to mechanical reinforcement, its lower mechanical performance suggests that MEF alone may require additional reinforcement (e.g., by converging it with MEW or complementary reinforcing strategies) to provide sufficient mechanical support for a cartilage implant. Still, as both MEW and MEF scaffolds are composed of PCL, their mechanical contribution is expected to gradually diminish as the polymer undergoes slow degradation in vivo.

By using an accelerated lipase assay as an in vitro approximation of the long‐term degradation process, we evaluated scaffold degradation (Figure S4). MEW and MEF scaffolds showed comparable degradation behavior over time, suggesting that the different fabrication approaches did not substantially alter the degradation tendency of PCL. Importantly, this highlights that the long‐term functionality of these constructs will depend not only on the initial mechanical reinforcement provided by the scaffolds, but also on the ability of cells to progressively deposit and mature their own ECM. Therefore, in the following sections, ECM deposition was evaluated to further assess the long‐term translational potential of MEW‐ and MEF‐based cartilage constructs.

Finally, sessile drop contact angle measurements indicated that the different scaffolds (Neat PCL, MEW PCL, Pre‐MEF PVAc/PCL, and MEF PCL) exhibit comparable wettability, with only minor variations observed across conditions (Figure S5). These differences were primarily attributed to variations in fiber topography and surface architecture rather than material chemistry. However, these measurements were performed under dry conditions and therefore do not fully represent the hydrated environment experienced by cells during culture. Thus, while the relatively similar contact angles suggest that differences in dry‐state wettability alone are unlikely to fully explain the later observed cellular responses, a contribution of surface wettability under hydrated conditions cannot be excluded.

### 2.4 MEF PCL for Cell Alignment and Distribution

Following optimization of fabrication parameters, pre‐MEF PVAc/PCL was fibrillated through selective dissolution of PVAc in 70% (*v/v*) ethanol, followed by subsequent washing in phosphate‐buffered saline (PBS). MEW PCL scaffolds were subjected to the same treatment to ensure comparable evaluation. Articular cartilage progenitor cells (ACPCs) were cultured on both scaffold types to assess whether MEF scaffolds enhanced cell alignment and organized deposition of ECM compared to MEW PCL.

First, to enable controlled cell‐fiber interaction, a custom platform was developed in which a single fiber could be printed, seeded with ACPCs, and non‐adhered cells subsequently removed. This system allowed direct cell attachment comparison between a single MEF PCL nanofibrillar bundle and a single MEW PCL microfiber (Figure 4A). The cell seeding density was optimized, with 5 × 10^6^ cells/mL identified as optimal for consistent initial coverage across both fiber types (Figure 4B). ACPCs readily attached to both substrates; however, MEF PCL bundles promoted a more uniform cell coverage along the fiber, whereas on MEW PCL, cells showed a tendency to aggregate (Figure 4B,C). Quantification of total DNA content at day 1 after cell seeding further demonstrated significantly higher initial cell attachment on MEF PCL compared with MEW PCL scaffolds (Figure S6).

**FIGURE 4:**
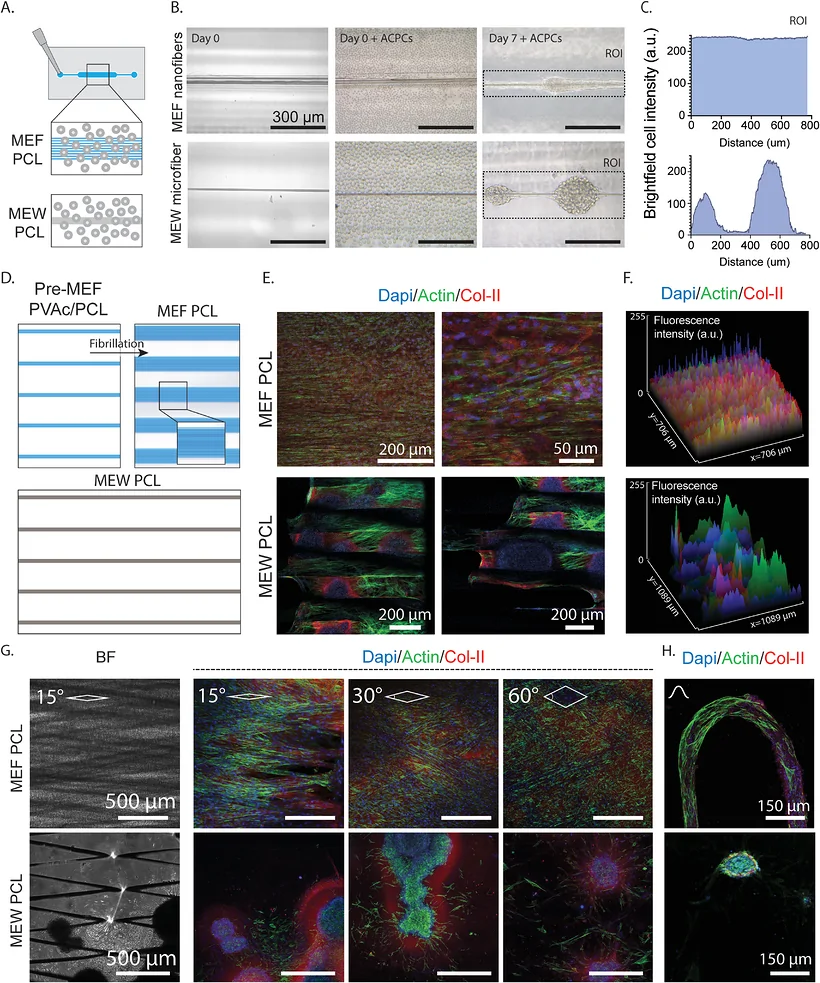
Comparison of ACPC behavior on MEF PCL nanofibrillar and MEW PCL microfiber scaffolds. (A) Schematic overview of the chip designed to study individual MEF nanofibril bundles and MEW microfibers. (B) Brightfield images of ACPC attachment after 7 days of culture (scale bar: 300 µm) are shown in all images. (C) Brightfield intensity profile along the region of interest (ROI) indicated in B, corresponding to the representative image shown. (D) Schematic representation of pre‐MEF PVAc/PCL scaffolds before and after fibrillation (top), compared to MEW PCL constructs (bottom), highlighting post‐fibrillation MEF fiber expansion. (E) Representative microscopy images showing cell alignment and ECM deposition (blue = DAPI/nuclei; green = actin; red = collagen type II). (F) 3D surface plots representing spatial fluorescence intensity distribution for DAPI, actin, and collagen across the scaffold surface, corresponding to the representative image in 4E. (G) Representative brightfield and fluorescence images of ACPC‐laden rhombus‐shaped scaffolds with angles ranging from 15° to 60° (scale bar: 500 µm; blue = DAPI/nuclei; green = actin; red = collagen type II). (H) Representative fluorescence images of cell‐laden MEF PCL and MEW PCL curved scaffolds (blue = DAPI/nuclei; green = actin; red = collagen type II).

Next, ACPCs were chondrogenically differentiated for 28 days on unidirectionally printed constructs of MEW and MEF PCL (Figure 4D and Figure S8). MEF constructs exhibited uniform cellular coverage, with actin filaments aligned along the nanofibril bundle axis and collagen deposition following the printed architecture (Figure 4E and Figure S7). In contrast, MEW PCL scaffolds showed cell aggregation within inter‐fiber spaces (Figure 4E and Figure S7). These findings were consistent with the observations from single‐fiber interactions. The increased surface area and reduced effective pore size of MEF scaffolds following fibrillation (even when initially printed with the same spacing as MEW PCL scaffolds) may further contribute to the more homogeneous cell distribution, although the individual contributions of surface area, pore size, and nanofibrillar topography cannot be separated in this comparison. Smaller pore sizes could not be achieved with MEW PCL due to limitations in stacking fidelity, which remains a constraint. Yet, MEF PCL provided strong guidance for cell alignment and uniform ECM distribution (Figure 4F).

Moreover, rhombus‐shaped PCL scaffolds with angles of 15°, 30°, and 60° were used to evaluate the influence of scaffold geometry on cell–material interactions (Figure 4G; Figures S7 and S8). ACPCs cultured on MEF PCL rhombus scaffolds exhibited consistent alignment along the designed fiber orientations, with a homogeneous distribution of F‐actin across the scaffold surface (Figure S9A). Quantitative analysis confirmed higher F‐actin spatial homogeneity on MEF scaffolds, whereas MEW PCL scaffolds exhibited greater spatial heterogeneity, consistent with the pronounced cell clustering observed across all geometries (Figure S9B). Furthermore, orientation analysis revealed distinct F‐actin alignment peaks corresponding to the underlying PCL fiber orientations in MEF scaffolds, whereas no clear directional alignment was observed for cells on MEW scaffolds (Figure S9C).

To further assess cell behavior in non‐linear geometries, individual fiber‐curved structures with sinusoidal wavelengths of 1 and 3 mm were fabricated (Figure S10). As these experiments were performed on individual fibers rather than porous scaffolds, differences in scaffold‐level pore size or porosity do not contribute to the comparison. MEF PCL nanofibrillar scaffolds supported continuous cell adhesion and alignment along the curved paths. In contrast, cells on MEW PCL scaffolds did not follow the imposed curvature and instead aggregated at the peaks of the arches (Figure 4H and Figure S11). Together with the single‐fiber attachment results (Figure 4A–C), these findings indicate that the altered fiber‐scale topography of MEF PCL can promote more continuous cell attachment and guidance independently of scaffold‐level pore architecture.

The ability of cells to follow both linear and non‐linear (angled and curved) nanofibrillar features highlights the potential of MEF PCL as a topographical cue for guiding cell distribution. However, the enhanced cellular organization observed with MEF PCL cannot be attributed exclusively to fiber diameter, as fibrillation simultaneously alters several interrelated architectural features, including nanofibrillar surface topography, effective surface area, fiber density, and pore size. These parameters were not independently controlled and may collectively contribute to the observed differences in cell coverage and alignment. Importantly, the single‐fiber experiments minimize the contribution of scaffold‐level pore size and demonstrate more uniform cell coverage on individual MEF fiber bundles, supporting a contribution of the distinct nanofibrillar fiber topography to cell–scaffold interactions. Nevertheless, the relative contributions of nanofibril dimensions, surface area, pore architecture, and cell retention cannot be independently determined from the present experiments.

To further investigate the mechanisms underlying cell alignment in MEF PCL scaffolds, we performed an additional experiment (Figure S12) using half‐cylindrical substrates with defined diameters (250–1500 µm), either uncoated or overlaid with perpendicularly aligned MEF PCL fiber bundles. On uncoated control cylinders, ACPCs aligned along the cylinder axis only at diameters ≤ 500 µm, consistent with curvature‐driven alignment, where cells tend to minimize bending of their actin cytoskeleton along directions of high curvature [52]. In contrast, when cultured on MEF fibers, ACPCs consistently aligned along the fiber direction regardless of cylinder curvature or fiber orientation, indicating that the MEF fiber architecture can provide a strong directional cue that overrides the mesoscale curvature cue under these conditions.

This pronounced guidance effect is likely related to the distinct anisotropic surface architecture of the MEF PCL bundles. Directional cell migration through contact guidance is known to occur when cells align and migrate along anisotropic, structured features. Individual MEF fibrils (∼500 nm in diameter) are smaller than the cell size and, although somewhat larger than native collagen fibrils, their arrangement creates interfibrillar “voids” with spacings on the order of hundreds of nanometers to a few micrometers (mean ∼850 nm), comparable to native collagen fiber spacing. Such features are known to promote strong contact guidance [53].

### 2.5 Chondrogenesis on High‐Density Arched MEF PCL Scaffolds

ACPCs cultured on MEF PCL scaffolds exhibited a pronounced elongated morphology along the fiber direction. While such elongation has been associated with chondrocyte dedifferentiation [54], the elongation of cartilage progenitor cells, known for their migratory behavior in response to injury or chemotactic signals in vivo, does not necessarily lead to dedifferentiation provided that chondrogenic matrix synthesis is maintained [55, 56]. For this reason, the deposition of cartilage‐specific ECM components, including collagen type II and glycosaminoglycans (GAGs), was studied.

Large‐scale, high‐density MEF PCL constructs were fabricated to mimic the tightly arched collagen fiber architecture characteristic of native articular cartilage (Figure 5A–C). Following fibrillation, the nanofibrillar scaffolds were seeded with ACPCs and cultured under chondrogenic conditions for 28 days. Picrosirius red staining showed a dense collagen network formation (Figure 5D). Collagen type II deposition was confirmed by immunohistochemistry, while Safranin‐O staining demonstrated an abundant and homogeneous distribution of GAGs throughout the scaffold (Figure 5E), confirming the formation of a cartilage‐like ECM. Quantitative analysis for GAGs and for total collagen demonstrated a progressive increase in both matrix components over the 28‐day culture period (Figure S14).

**FIGURE 5:**
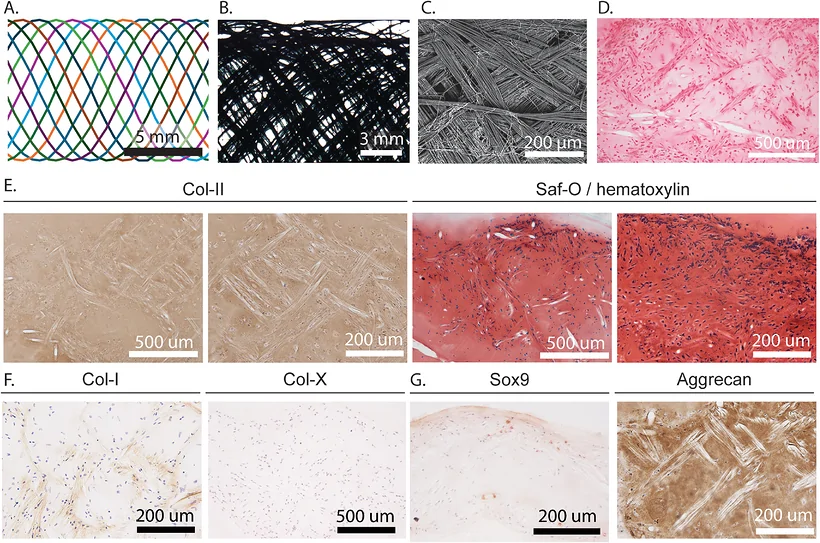
Chondrogenesis on high‐density MEF PCL arched scaffolds. (A) Schematic representation of the arched print pattern, modeled as a sinusoidal wave function. (B) Brightfield image of the arched MEF PCL network following fibrillation; scale bar: 3 mm. (C) SEM image of the fibrillated high‐density arched network; scale bar: 200 µm. (D–G) Staining of ACPC‐seeded constructs after 28 days of chondrogenic differentiation; representative of *n* = 3 independent scaffolds (single donor). (D) Picrosirius red staining; scale bar: 500 µm. (E) Collagen type II (immunohistochemistry) and Safranin‐O (sulfated GAGs); scale bar: 500 µm (left) and 200 µm (right). (F) Collagen types I and X (immunohistochemistry); scale bar: 200 and 500 µm, respectively. (G) SOX9 and aggrecan core protein (immunohistochemistry); scale bar: 200 µm.

Collagen type I staining revealed minor deposition (Figure 5F), consistent with in vitro ACPC chondrogenesis [25, 57, 58]. Importantly, no detectable collagen type X deposition (Figure 5F) indicated the absence of hypertrophic differentiation, also consistent with the tendency of ACPCs to be less prone to hypertrophy than other cell sources [55, 58]. Consistent with active chondrogenesis, positive nuclear staining for the transcription factor SOX9 was observed in a subset of cells (Figure 5G), likely induced by TGF‐β supplementation (10 ng/mL) in the differentiation medium. Aggrecan deposition was further confirmed by staining of its core protein (Figure 5G). This observation is in line with safranin‐O staining, which binds to negatively charged glycosaminoglycan chains (chondroitin sulfate and keratan sulfate), corroborating the presence of a proteoglycan‐rich ECM.

### 2.6 Collagen Alignment on MEF PCL Scaffolds

Histology predominantly indicated the presence of collagen type II within these constructs (Figures 4 and 5). However, matrix deposition does not necessarily imply collagen alignment. To assess fibrillar orientation, multiple complementary techniques were used.

Multiple‐angle acquisition polarized light microscopy (PLM) was used to image picrosirius red‐stained samples for visualization of collagen alignment. Collagen, together with the anionic picrosirius red dye bound to it, exhibited birefringent signals tracing the sinusoidal paths of the MEF scaffold (Figure 6A,B). Notably, no residual PCL signal was expected, as the polymer dissolves during histological processing in xylene. Quantitative orientation analysis revealed two distinct peaks around −45° and 45°, corresponding to the slopes of the sinusoidal architecture (Figure 6C,D), thereby indicating that the scaffold geometry guided the directional organization of the deposited collagen matrix.

**FIGURE 6:**
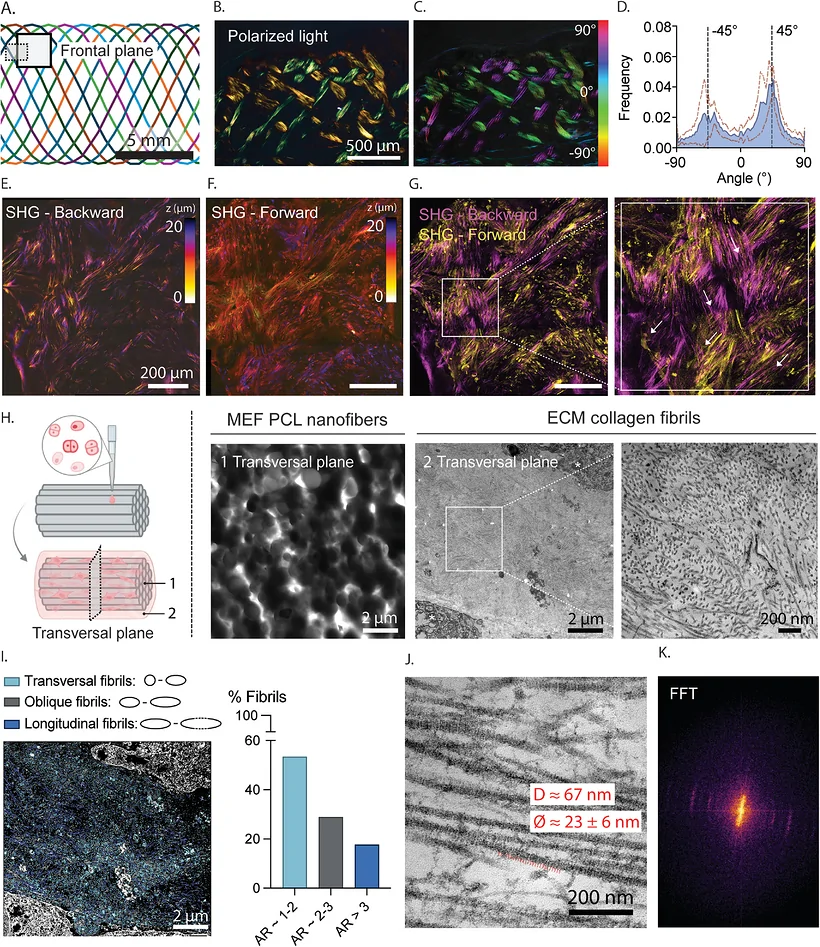
Collagen organization and alignment on MEF PCL arched scaffolds. (A) Schematic representation of the arched print pattern, modeled as a sinusoidal wave function; scale bar: 5 mm. (B) PLM maximum projection image (rotatable analyzer at 0°, 22.5°, and 45°); scale bar: 500 µm. (C) Color‐coded collagen orientation map (−90° to 90°); scale bar: 500 µm. (D) Quantitative orientation distribution of collagen, pooled from *n* = 3 independent scaffolds, with SD in red, showing two peaks at −45° and 45°, corresponding to the slopes of the MEF PCL arches. (E) Backward‐detector SHG, color‐coded for depth (0–20 µm); scale bar: 200 µm. (F) Forward‐detector SHG, color‐coded for depth (0–20 µm); scale bar: 200 µm. (G) Overlay of backward (pink) and forward (yellow) SHG signals, zoomed to show the crisscrossed fibril architecture; scale bar: 200 µm. (H) Schematic representation of TEM imaging planes with micrographs: (1) central MEF PCL bundle (scale bar: 2 µm) and (2) cells and surrounding ECM (scale bar: 2 µm), sectioned in the transverse plane, with a higher magnification inset (scale bar: 200 nm); cells indicated with an asterisk (*). (I) Aspect‐ratio analysis of collagen fibrils in a representative micrograph from the transverse section (TEM from 2 scaffolds in complementary orientations; 13 775 fibrils quantified within the field); scale bar: 2 µm. (J) Zoom‐in of a collagen fibril showing D‐periodic banding (∼67 nm) and average diameter 23 ± 6 nm (*n* = 10); scale bar: 200 nm. (K) Fast Fourier transform (FFT) of a collagen fibril, showing the first harmonic corresponding to D‐periodicity ∼67 nm.

Next, to confirm the visualization of collagen fibrils, second harmonic generation (SHG) microscopy was used, intrinsically specific for non‐centrosymmetric fibrillar collagen. Forward SHG (signal collected along the laser path) is more sensitive to fibers with an axial component, whereas backward SHG (signal collected toward the source) is more sensitive to fibers in the lateral plane. For both cases, collagen was detected organized in a crossed pattern, following the underlying arched PCL structure across the sample depth (Figure 6E–G). This was accompanied by alignment of the cellular F‐actin cytoskeleton along the scaffold architecture (Figure S15).

Lastly, samples were processed for transmission electron microscopy (TEM) to directly visualize the ultrastructural organization of the deposited collagen. MEF PCL scaffolds seeded with ACPCs and chondrogenically differentiated for 28 days were sectioned in the transverse plane (Figure 6H). On the micrographs, fibrillar bundles of the MEF PCL appeared cross‐sectioned (Figure 6H‐1), transverse to the cutting plane, in a tightly aligned packing above a mix of cells and a collagen‐rich ECM (Figure 6H‐2). Collagen fibrils between cells (*) were also predominantly sectioned transversely, indicating alignment along the longitudinal axis of the PCL scaffold. To quantify this observation, aspect ratio (AR) measurements were used to distinguish between transverse (low AR) and longitudinal (high AR) orientation. Approximately 53% of fibrils exhibited an AR between 1 and 2, consistent with a more transverse orientation, whereas only 17% displayed an AR > 3 (Figure 6I). TEM images also confirmed the fibrillar nature of the collagen, revealing the characteristic D‐banding pattern of native collagen fibrils (first harmonic of ∼67 nm measured by Fast Fourier Transform, FFT) and an average fibril diameter of 23 nm (Figure 6J,K), in line with that observed in vivo [59].

These analyses collectively demonstrate that MEF PCL scaffolds effectively guide collagen alignment along the fabricated architecture, as consistently evidenced by PLM, SHG, and TEM observations (Figure 6). Quantitative assessments of orientation and aspect ratio further confirmed that collagen fibrils are predominantly aligned with the scaffold axis. This indicates that the deposited ECM is able, to a considerable extent, to recapitulate the alignment dictated by the underlying MEF PCL scaffold. Notably, this represents a notable advancement, as achieving controlled alignment of collagen has remained a major challenge in cartilage tissue engineering. However, within the timeframe of our in vitro experiments, no substantial PCL degradation was observed. As a result, the specific mechanical contribution of the highly aligned collagen network could not yet be decoupled from that of the PCL scaffold itself.

The question therefore arises as to what drives the alignment of the deposited collagen along the MEF architecture. Two complementary, non‐mutually exclusive mechanisms can be considered. First, it is well established across connective‐tissue models that cells which become aligned on anisotropic fibrous or grooved substrates deposit a correspondingly aligned collagenous matrix, with cell alignment typically preceding and determining the formation of aligned collagen‐rich ECM [60, 61]. This has been mechanistically linked to spatially constrained focal adhesion maturation and F‐actin polarization along the substrate, generating anisotropic traction forces that bias cell orientation and directional migration along the aligned topography [62, 63]. It is important, however, to qualify how far an active, cell‐mediated mechanism can be invoked in a cartilage context. In native articular cartilage, the current consensus does not support an active fibripositor‐mediated secretion of aligned collagen type II by chondrocytes [64]; unlike, for example, in embryonic tendon fibroblasts, where active fibril secretion has been described for collagen type I, an equivalent active guidance mechanism has not been demonstrated for chondrocytes. Instead, cartilage collagen organization is increasingly proposed to arise from a largely passive, extracellular phenomenon, in which collagen type II fibers anchored at the subchondral bone are pulled into alignment by directional interstitial tissue growth, and growth from within the tissue is required to obtain aligned collagen [18, 65, 66]. This distinction is relevant here because mature chondrocytes in native 3D cartilage are typically rounded, encapsulated within a pericellular matrix, and considered unable to directly reorganize collagen fibers. The ACPCs used in this study (more progenitor‐like and migratory), however, are contact‐guided and elongated along the MEF fibers, so a contribution of cell‐shape‐mediated deposition cannot be excluded. We therefore do not attribute the observed collagen alignment to an active cellular fibril‐secretion mechanism, which remains unproven for chondrocytes, but interpret it as most parsimoniously explained by a passive, template‐driven process: the anisotropic MEF fibrillar architecture, including its inter‐fibrillar voids and high surface area, provides the directional physical template that biases collagen deposition along the fiber axis with a possible additional contribution from the elongated, contact‐guided ACPC morphology. This framing is also consistent with our half‐cylinder experiment (Figure S12), in which the MEF fiber cue overrode mesoscale curvature‐driven alignment, supporting the scaffold's anisotropy as the dominant directional template. Although the individual MEF fibrils are larger than native cartilage collagen type II fibrils (∼30–200 nm), MEF PCL bundles operate at the cell scale as anisotropic guidance structures [67]. We therefore propose that MEF PCL reproduces an organizing principle of native cartilage collagen networks rather than directly mimicking collagen fibril dimensions.

Beyond establishing this initial alignment, an important consideration is whether the newly deposited collagen type II network will retain it as the MEF PCL scaffold degrades. Previous work in anisotropic electrospun PCL scaffolds under mechanical constraint has shown that low‐rate scaffold degradation preserved contact‐guided collagen orientation, whereas high‐rate degradation allowed the still‐immature collagen network to reorient toward the constraint direction, indicating that newly deposited collagen initially lacks sufficient self‐templating capacity [68]. Consistent with this, we observed no substantial PCL degradation over our 28‐day culture, in line with the reported 12–48‐month in vivo degradation timescale of PCL, and a short‐term (4‐day) accelerated lipase assay on neat MEW and MEF PCL fibers showed no measurable difference in degradation between the two fiber scales [69]. This is a further advantage of the MEF architecture: it already achieves superior collagen orientation over MEW microfibers without introducing an additional degradation liability, even though the two do not appear to differ in short‐term degradation rate. These findings potentially place our constructs in a low‐degradation regime associated with alignment maintenance, but the longer‐term persistence of MEF‐templated collagen anisotropy in vivo will depend on whether the collagen network matures sufficiently before PCL degradation becomes substantial [68].

## 3 Conclusions and Outlook

In this study, we demonstrate that MEF enables the fabrication of PCL scaffolds combining microscale architectural control with a nanofibrillar surface topography. The resulting MEF architecture provides effective directional cues for ACPC alignment and organized collagen‐rich ECM deposition, extending the architectural and topographical capabilities of conventional MEW PCL scaffolds. Overall, our findings support the hypothesis that the anisotropic MEF architecture can guide the organization of newly deposited collagen, while the relative contribution of the underlying topographical and cell‐mediated mechanisms remains to be determined.

Longer‐term in vivo studies will be needed to determine whether collagen organization is maintained as PCL gradually degrades and whether the developing ECM progressively contributes to the mechanical function of the construct. Future work should focus on translating this architectural guidance into mechanically functional tissue. Integration of MEF with mechanically reinforcing MEW structures could enable larger, load‐bearing constructs while retaining the nanofibrillar guidance cues provided by MEF.

Overall, this work establishes MEF as a versatile biofabrication approach for introducing controlled nanofibrillar topographical cues within predefined three‐dimensional architectures, providing a promising platform for engineering structurally organized tissues.

## 4 Study Limitations

Several limitations of the present study should be acknowledged. First, the current MEF process involves preparation of the PCL/PVAc blend in dichloromethane (DCM), a volatile and toxic solvent requiring careful handling and removal. Although DCM use is not inherent to MEF and solvent‐free PCL/PVAc melt blending has been previously demonstrated, its use introduces practical and translational considerations [35]. The use of PCL/PVAc also reflects a trade‐off between the thermal processability and mechanical tunability required for MEF and the limited compatibility of natural polymers, such as collagen, gelatin, and chitosan, due to the elevated processing temperatures. In addition, PVAc removal reduced scaffold cohesion and increased brittleness, while increasing layer number reduced stacking efficiency, and low‐angle rhombic geometries showed reduced printing fidelity. For applications requiring larger or load‐bearing constructs, these limitations may therefore need to be addressed through further process optimization or by combining MEF with complementary reinforcement strategies.

Second, MEF and MEW scaffolds differ in several interconnected architectural features beyond fiber diameter, including effective surface area, fiber density, and pore size in bigger scaffolds. These parameters were not independently controlled, and therefore their individual contributions to cell attachment, alignment, and ECM organization cannot be separated. Single‐fiber experiments minimized the contribution of scaffold‐level pore size and supported a role for fiber‐scale MEF topography, but the relative contribution of individual architectural features remains unresolved.

Third, contact‐angle measurements were performed under dry conditions and therefore do not directly represent the fully hydrated cellular environment. These measurements should consequently be interpreted as a comparative assessment of dry‐state wettability.

Finally, although organized, collagen type II‐rich ECM deposition was demonstrated, our short‐term in vitro setup could not decouple the mechanical contribution of the aligned collagen network from that of the intact PCL scaffold, nor determine how this network would behave once PCL degradation becomes substantial. Degradation itself was characterized separately in an accelerated in vitro assay, but combining substantial scaffold degradation with a cell‐seeded, ECM‐rich construct while preserving the newly deposited network was not feasible in the current setup. It should also be determined whether scaffold deformation during tissue development affects the persistence of the predefined anisotropic cues and collagen organization. This represents an important direction for future in vivo studies.

## 5 Methods

### 5.1 Printing Materials

A polymer blend (pre‐MEF PVAc/PCL) composed of 70 wt% poly(vinyl acetate) (PVAc; Kremer Pigments GmbH & Co. KG, Germany) and 30 wt% polycaprolactone (PCL; PC‐12, Corbion, The Netherlands) was prepared by dissolution in DCM under continuous stirring for 1 h. All procedures involving DCM were conducted in a chemical fume hood due to its volatility and toxicity. Following complete dissolution, DCM was allowed to evaporate under ambient conditions in the fume hood. The resulting polymer blend was subsequently vacuum‐dried to ensure complete removal of residual solvent, yielding a solvent‐free material suitable for printing [35]. Blend preparation was carried out at 21°C and 35% relative humidity. For the MEW PCL samples, neat PCL (PC‐12 Corbion, The Netherlands) was used without further modification.

### 5.2 Scaffold Design and Fabrication

#### 5.2.1 MEF

The pre‐MEF PVAc/PCL polymer blend was processed using an in‐house built MEW system. The blend was heated to 170°C in a 3 mL glass syringe and extruded through a 25G nozzle under an applied voltage of 3–6 kV and air pressure of 0.5–2 bar. The collector distance was set to 3 mm [35, 36, 42]. Due to its higher viscosity compared to neat PCL, the PVAc/PCL blend required elevated processing to achieve a stable jet and consistent fiber deposition. Fibers were deposited onto glass slides mounted on the collector plate, which translated in the X‐ and Y‐ directions with a collector velocity ranging from 2 to 12 mm/s. Motion control was achieved using custom‐written scripts in Motion Perfect (Trio Motion Technology, UK). Post‐processing fibrillation of the PVAc/PCL printed fibers was achieved by selective removal of PVAc. Samples were immersed in 70% (*v/v*) ethanol (3 × 10 min) to dissolve the PVAc phase, followed by rinsing in sterile PBS (3 × 5 min) to remove residual polymer and solvent. This process yielded bundles of PCL nanofibrils (referred to as MEF PCL in the manuscript).

#### 5.2.2 Melt Electrowritten PCL Scaffolds

Melt electrowritten PCL (MEW PCL) control scaffolds were fabricated using the same in‐house‐built printer. PCL (PC‐12, Corbion, The Netherlands) was heated to 100°C in a 3 mL glass syringe and extruded through a 24G nozzle under an applied voltage of 5–6 kV, a pressure of 1 bar, and a collector distance of 3 mm. Fibers were deposited onto a translating collector moving in the *X*‐ and *Y*‐directions at a constant speed of 8 mm/s, controlled via custom‐written scripts in Motion Perfect (Trio Motion Technology, UK).

Both MEW and MEF are melt‐based fabrication techniques optimized for thermoplastic polymers and the generation of reinforcement scaffolds. As such, these processes are not compatible with natural polymers (e.g., collagen, gelatin, or chitosan), which undergo thermal denaturation under the elevated processing temperatures required for melt processing.

#### 5.2.3 Scaffolds With Box and Rhombus Architectures

Scaffolds with box‐shaped architectures were fabricated by MEW using both neat PCL and the PVAc/PCL blend, with an inter‐fiber spacing of 300 µm. Constructs were printed with thicknesses of 1, 10, 25, and 50 layers. In addition, rhombus‐shaped architectures were generated by varying the fiber orientation angles to 15°, 30°, and 60°.

#### 5.2.4 Single Line MEW PCL and MEF PCL

Single fibers of MEW PCL and pre‐MEF PVAc/PCL polymer blend were printed onto rectangular glass coverslips. Microfluidic devices were fabricated using negative molds of PIC‐100 produced via digital light processing (DLP) 3D printing (EnvisionTEC P4K). Polydimethylsiloxane (PDMS; Sylgard 184, Farnell) was cast onto the molds and cured overnight at 60°C to obtain chips containing a central perfusion channel. Following fabrication, the PDMS chips and glass coverslips were plasma‐treated and bonded. For MEF samples, the embedded pre‐MEF PVAc/PCL fibers were subjected to the fibrillation process described above to remove PVAc and expose the underlying MEF PCL nanofibrils.

#### 5.2.5 Scaffolds With Arched Architectures

Single‐layer sinusoidal arched structures were fabricated using both MEW and MEF, with wavelengths of 1 and 3 mm. To stabilize the prints, a PCL frame was fabricated via fused deposition modeling (FDM; 3D Discovery, RegenHU) using an extrusion temperature of 80°C, a feed rate of 7 rev/min, and a collector velocity of 5 mm/s. The frame was secured to glass coverslips using PDMS, enabling stabilization of the arch structures during and after printing. For high‐density arched architectures, a MEW base layer with a fiber spacing of 100 µm was first deposited. Subsequently, sinusoidal arch structures were printed with a period of 4π mm, an amplitude of 4 mm, and a total width of 20 mm. Each layer consisted of 12 sinusoidal waves with a phase shift of π/12. After every two layers (24 waves), three additional horizontal MEW layers were deposited to enhance mechanical stability. This sequence was repeated 12 times, resulting in a total of 288 sinusoidal waves, forming a high‐density fibrillar network. The resulting constructs were therefore hybrid MEF/MEW scaffolds, in which the MEW fibers served solely as a supporting framework to maintain the stability of the three‐dimensional architecture during fabrication and handling. The fibrillar network investigated throughout this study was formed by the MEF‐printed sinusoidal arches.

### 5.3 Cell Isolation, Culture Conditions, and Cell Seeding

Equine articular cartilage chondroprogenitor cells (ACPCs) were isolated from the healthy knee joints of a juvenile (<1 year) equine donor (Equus ferus caballus) via enzymatic digestion using 0.2% pronase for 2 h, followed by overnight digestion with 0.075% collagenase II [26, 58]. All procedures were conducted in accordance with the guidelines of the Ethical and Animal Welfare Committee of Utrecht University. ACPCs were selected based on fibronectin adhesion (10 µg/mL) and colony formation, and expanded in growth medium consisting of DMEM high glucose with GlutaMAX and pyruvate (Gibco, 31966), supplemented with 10% fetal bovine serum (FBS; Westburg, CA FBS‐HI‐11A), 1% penicillin/streptomycin (Gibco, 15140‐122), 1% ascorbic acid 2‐phosphate (Merck, A8960), 1% non‐essential amino acids (Gibco, 11140‐050), and 5 µL/mL basic fibroblast growth factor (bFGF; Bio‐Techne, 233‐FB), up to passage 3. Cells were subsequently seeded onto MEW PCL or MEF PCL scaffolds after fabrication and sterilization, under physiological conditions. Constructs were cultured in chondrogenic differentiation medium consisting of DMEM high glucose with GlutaMAX and pyruvate (Gibco, 31966), supplemented with 1% ITS Premix (Corning, 354352), 1% penicillin/streptomycin (Gibco, 15140‐122), 1% ascorbic acid 2‐phosphate, 1% HEPES buffer (1 M; Gibco, 15630‐080), 0.1 µM dexamethasone (Sigma–Aldrich, D8893), and 10 ng/mL recombinant human transforming growth factor‐β1 (TGF‐β1; PeproTech, 100‐21). Medium was refreshed twice per week for up to 28 days under standard culture conditions (37°C, 5% CO_2_) [26].

### 5.4 Analysis

#### 5.4.1 Fibril and Scaffold Characterization

Scaffolds were imaged using stereomicroscopy (Olympus SZ61), bright‐field microscopy (Olympus BX53), and scanning electron microscopy (SEM; Phenom Pro Desktop SEM, Thermo Fisher Scientific, USA). SEM imaging was performed at an accelerating voltage of 5–10 kV to visualize the MEF PCL and MEW PCL fibers. No conductive metal coating was applied, as the scaffolds provided sufficient contrast for imaging. Fiber diameter, orientation angle, wavelength, and scaffold height were measured with ImageJ software (version 1.54f). To assess deposition accuracy, a subset of the box‐shaped pores from the central region of each scaffold was analyzed. Accuracy was calculated by comparing the measured pore area to the corresponding theoretical value [70].

#### 5.4.2 Mechanical Characterization

The compressive and tensile properties of the scaffolds were assessed using a dynamic mechanical analyzer (DMA; Q800 TA Instruments). Measurements were carried out at 21°C and 50% relative humidity. To evaluate the reinforcement capacity of the fibers, compression testing was performed on MEF and MEW scaffolds (inter‐fiber spacing: 300 µm; height: 1 mm) embedded in 10% w/v gelatin methacryloyl (gelMA), prepared as previously described [71]. Crosslinking was achieved using 0.5 mM dichloro‐ruthenium (II) hexahydrate and 5 mM sodium persulfate (both from Sigma–Aldrich, USA), followed by exposure to a 20 W LED floodlight (Jobmate, Australia) for 7 min at a distance of 7 cm. Unconfined compression testing was conducted with a preload of 0.001 N, followed by a controlled strain rate of −20% per minute up to −30%, while the applied force was recorded. Applied stress was calculated, and the elastic compressive modulus was determined from the linear region of the stress‐strain curve (10%–15% strain). Cyclic compression testing was performed using a strain‐controlled protocol. Samples were subjected to cyclic compression between 10% and 30% strain at a strain rate of 20% strain/min, with each cycle lasting 1 min, for a total of five cycles while the stress and strain were recorded. Following the final cycle, the samples were unloaded and allowed to recover for 1 min. The dynamic modulus was calculated by determining the E‐modulus for each individual slope and combining these values. Stress‐relaxation testing was performed under a constant compressive strain of 20% following a preload of 0.01 N. Samples were displaced to −20% strain over 2 min and subsequently held at this strain to monitor the time‐dependent decrease in stress. Following the relaxation period, the displacement was returned to 0%, and the samples were allowed to recover for 1 min. The relaxation modulus was normalized to the maximum modulus measured for each sample, and the normalized modulus at the end of the relaxation period was used to calculate the elastic index.

For tensile testing, MEF PCL and MEW PCL scaffolds (inter‐fiber spacing: 300 µm; dimensions: 30 x 10 mm) were mounted in a cardboard frame and secured in tensile clamps to prevent any damage to the scaffold during handling. The frame was then cut prior to testing. Samples were subjected to a controlled strain rate of 25% per minute up to 50% strain, while force was recorded. Stress was calculated, and the elastic tensile modulus was determined from the linear region of the stress‐strain curve (1%–5% strain).

#### 5.4.3 Material Characterization

##### 5.4.3.1 Fourier Transform Infrared Spectroscopy

Fourier transform infrared (FT‐IR) spectroscopy was used in attenuated total reflectance (ATR) mode to take spectra of the samples. The samples were prepared by dissolving them in DCM at 20% (*w/v*), and subsequently casting them into glass Petri dishes. The solvent was allowed to evaporate in a fume hood overnight to yield the final thin films measured. Spectra were acquired using an FT‐IR spectrometer (Frontier, PerkinElmer). FT‐IR spectra were recorded over a wavenumber range of 4000–550 cm^−^^1^ at a resolution of 1 cm^−1^, averaging 4 scans per sample. Characteristic absorption peaks corresponding to PVAc and PCL were identified and compared to confirm the selective removal of PVAc following processing.

##### 5.4.3.2 Mass Loss Analysis

To quantify mass loss associated with PVAc removal during fibrillation, pre‐MEF PVAc/PCL constructs were weighed prior to treatment to determine their initial mass. Constructs were then subjected to the fibrillation procedure described above, while control constructs underwent the same handling conditions in PBS without fibrillation. Following treatment, constructs were collected and weighed again after removal of excess liquid. The percentage mass loss was calculated relative to the initial construct mass according to: Mass loss (%) = [(initial mass−final mass)/initial mass] × 100.

##### 5.4.3.3 Tensiometry

Surface wettability was assessed using a contact angle measurement system (OCA 15EC, DataPhysics Instruments). Prior to analysis, samples were rinsed in 70% (*v/v*) ethanol followed by Milli‐Q water and allowed to dry to ensure all samples underwent the same treatment. Samples were then mounted onto glass slides using single‐ or double‐sided adhesive tape. Static water contact angles were measured using the sessile drop method by depositing 0.2–0.5 µL droplets of Milli‐Q water onto the sample surface. Contact angles were determined using SCA20 software (DataPhysics Instruments) by averaging the left and right angles of each droplet. Each condition was measured in *n* = 3–9 replicates.

##### 5.4.3.4 Enzymatic Degradation

MEW and MEF PCL scaffold degradation was evaluated using lipase from Pseudomonas cepacia (62309, Sigma–Aldrich, USA). MEW and MEF scaffolds were incubated in PBS containing 0.25 mg/mL lipase and 0.1 mg/mL sodium azide (S2002, Sigma–Aldrich, USA), while PBS containing sodium azide without lipase served as the control [72]. Scaffolds were incubated individually at 37°C under gentle agitation, with the medium replaced daily. Samples were collected after 1, 2, 3, and 4 days. Following incubation, scaffolds were rinsed with Milli‐Q water, dried for 48 h at 37°C, equilibrated in a desiccator, and weighed. Mass loss was calculated relative to the initial dry mass.

#### 5.4.4 Biological Characterization for Functional Tissue Formation

##### 5.4.4.1 Live Brightfield and Whole‐Mount Fluorescence Microscopy

Brightfield images of the scaffolds were acquired using an inverted microscope (DMi1, Leica Microsystems). For fluorescence imaging, samples were first washed with PBS and fixed in 4% formaldehyde (VWR) for 30 min at room temperature. This was followed by permeabilization in PBS containing 0.3% Triton X‐100 (*v/v*). Samples were then blocked for 30 min with 5% bovine serum albumin (BSA; Merck) in PBS and incubated overnight at 4°C with a mouse primary antibody against collagen type II (DSHB Hybridoma II‐II6B3) at a dilution of 1:100. Following primary antibody incubation, samples were washed three times with PBS containing 0.1% Tween‐20 (PBS‐T) and incubated with a donkey anti‐mouse secondary antibody (Alexa Fluor 546, Thermo Fisher Scientific) for 1 h at room temperature. Samples were washed three times with PBS‐T again. Nuclear staining with DAPI (Sigma–Aldrich) and cytoskeletal staining with Alexa Fluor 488 phalloidin (Thermo Fisher Scientific) were then performed for 30 min at room temperature. Fluorescence imaging was carried out using a confocal laser scanning microscope (Leica SP8, Leica Microsystems).

##### 5.4.4.2 Metabolic Activity

Metabolic activity was assessed at days 1, 7, 14, and 21 of differentiation using an Alamar Blue assay (resazurin sodium salt, Alfa Aesar). A stock solution (11 ×) was prepared by dissolving resazurin in PBS at a concentration of 500 µM, followed by sterilization through a 0.22 µm filter. The working solution (1 ×) was obtained by diluting the stock in culture medium. Cell‐laden constructs, along with cell‐free controls, were transferred to a 48‐well plate, and 500 µL of the 1× Alamar Blue solution was added to each well. Samples were incubated for 4 h at 37°C in a humidified atmosphere with 5% CO_2_. Following incubation, 100 µL of the supernatant was collected and transferred to a black flat‐bottom 96‐well plate (Greiner). Fluorescence was measured using a microplate reader (CLARIOstar, BMG Labtech, Germany) at excitation/emission wavelengths of 560/590 nm (*n* = 6 biological replicates and *n* = 2 technical replicates per time point). After measurement, samples were returned to fresh culture medium. Fluorescence intensity was used as an indicator of metabolic activity.

##### 5.4.4.3 Biochemical Characterization

Glycosaminoglycan (GAG) content was quantified using a dimethylmethylene blue (DMMB) colorimetric assay (Sigma‐Aldrich, USA). Samples were collected after 1, 14, and 28 days of differentiation, freeze‐dried, and enzymatically digested overnight at 60°C in papain solution (7 U/mL). The GAG concentration was determined by measuring the absorbance of the DMMB–GAG complex at 520 nm using a microplate reader (CLARIOstar, BMG Labtech, Germany) (*n* = 6 biological replicates and *n* = 2 technical replicates per time point). Chondroitin sulphate standards were used to generate a calibration curve. Values were normalized to tissue dry weight to account for differences in sample size.

Total collagen content was assessed via hydroxyproline quantification using a colorimetric assay. Samples were digested in 0.4 M NaOH at 108°C overnight, followed by acid hydrolysis and reaction with chloramine T and p‐dimethylaminobenzaldehyde (DAB). Absorbance was measured at 560 nm using a microplate reader (CLARIOstar, BMG Labtech, Germany) (*n* = 6 biological replicates and *n* = 2 technical replicates per time point). L‐hydroxyproline (Merck) was used as a standard, and total collagen content was calculated using a hydroxyproline‐to‐collagen ratio of 1:7.5 [73, 74]. DNA content was quantified using a fluorometric assay (Quant‐iT PicoGreen dsDNA Assay kit, Invitrogen, USA), according to the manufacturer's instructions.

##### 5.4.4.4 Picrosirius Red and Safranin‐O/Fast‐Green Staining

Harvested constructs were fixed in 4% paraformaldehyde for 30 min at room temperature and dehydrated prior to paraffin embedding. Sections of 12 µm thickness were cut using a microtome (Leica). The sections were deparaffinized and stained with Weigert's hematoxylin for 10 min, followed by rinsing in water. For picrosirius red, samples were then stained with picrosirius red solution for 1 h, washed with 0.01 M hydrochloric acid, dehydrated with a series of alcohols, and mounted with permanent mounting medium (DePeX, 06522).

For safranin‐O/fast green staining, samples were stained with 0.4% *w/v* fast green solution for 4 min and rinsed with 1% *w/v* acetic acid. Upon washing, the samples were incubated with 0.125% *w/v* safranin‐O solution for 15 min, then washed with 70% ethanol (25 s), and subsequently dehydrated with 96% ethanol (2 ×), 100% ethanol (2 ×), xylene, and mounted with permanent mounting medium (DePeX, 06522).

##### 5.4.4.5 Immunohistochemistry

Paraffin‐embedded sections were first deparaffinized and washed in PBS. Endogenous peroxidase activity was quenched by incubating sections in 0.3% hydrogen peroxide in PBS for 10 min at room temperature. After three washes with PBS containing 0.1% Tween‐20 (PBS‐T), antigen retrieval was performed as follows. For collagen type I and II, sections were treated with 1 mg/mL pronase (from Streptomyces griseus, Merck) and 10 mg/mL hyaluronidase (from sheep testes, Type II, Sigma–Aldrich) for 30 min at 37°C. For collagen type X, slides were incubated with 1 mg/mL pepsin (from porcine gastric mucosa, Sigma–Aldrich) in 0.5 M acetic acid for 2 h, followed by 10 mg/mL hyaluronidase for 30 min at 37°C. For aggrecan, sections were alkylated with 40 mM iodoacetamide (Merk) for 2 h at room temperature and enzymatically treated with 25 mg/mL chondroitinase ABC (from Proteus vulgaris, Sigma–Aldrich) and 10 mg/mL hyaluronidase, both for 30 min at 37°C. Following antigen retrieval, sections were blocked in 5% bovine serum albumin (BSA, Millipore Sigma) in PBS for 30 min at room temperature and incubated overnight at 4°C with primary antibodies diluted in 5% BSA/PBS: collagen I (rabbit monoclonal, 1:100; Abcam, ab138492), collagen II (mouse monoclonal, 1:100; DSHB Hybridoma II‐II6B3), collagen X (mouse monoclonal, 1:30; eBioscience X53), aggrecan core protein (mouse monoclonal, 1:100; DSHB Hybridoma 12/21/1‐C‐6), and Sox9 (rabbit polyclonal, 1:100; Merck Millipore, AB5535). Sections were then washed three times with PBS‐T (10 min each) and incubated with the corresponding secondary antibodies (1:100; anti‐mouse IgG HRP or BrightVision+ anti‐rabbit IgG) for 1 h at room temperature. After washing (3 × 5 min in PBS‐T), staining was visualized using diaminobenzidine (DAB) chromogen (Agilent Technologies). Sections were counterstained with Mayer's hematoxylin, rinsed in water for 10 min, dehydrated through graded alcohols, and mounted with Eukitt mounting medium (Sigma–Aldrich). Images were acquired using an Olympus BX51 microscope.

### 5.5 Collagen Alignment Imaging

#### 5.5.1 PLM

Picrosirius red‐stained sections were imaged using an Olympus BX51 polarizing light microscope equipped with a rotatable analyzer, set at multiple angles (0°, 22.5°, and 45°), with the polarizer kept at a fixed angle. Brightfield images were acquired first to capture the general tissue morphology, followed by PLM images at each angle. At least three PLM images were acquired and merged using maximum intensity projection in ImageJ (version 1.54f). For quantitative analysis, images were converted to 8‐bit, and collagen fibril orientation was assessed using the OrientationJ plugin (version 2.0.5) and the Directionality plugin. Analyses were performed across the entire image without selecting a specific region of interest. Orientation data, including fiber direction and dispersion, were extracted and visualized as histograms, allowing assessment of both mean alignment and uniformity of collagen organization. This workflow follows established protocols for PLM analysis of picrosirius red‐stained collagen [75].

#### 5.5.2 Second Harmonic Generation (SHG) Microscopy

Second Harmonic Imaging was performed with a multiphoton microscope (TriMScope II, Miltenyi Biotec, Germany) equipped with a Ti‐Sapphire laser (Chameleon Ultra II, Coherent Inc., USA) and an Optical Parametric Oscillator (MPX OPO, Coherent Inc., USA) to generate excitation at 1230 nm with a power of approximately 65 mW under the microscope objective. Microscopy was performed using a 25 × 1.05 NA water‐immersion objective lens (XLPLN25XWMP2, Evident Inc., Japan). SHG emission was collected in the forward direction with a 1.4 NA condenser (Evident Inc., Japan), bandpass filtered with an ET620/60 (Chroma Technology GmbH, Germany), and detected using an alkali PMT (H7420‐20, Hamamatsu, Japan). Backward‐directed SHG emission was split from excitation light with a T900lpxxrxt‐UF2 longpass dichroic mirror (Chroma Technology GmbH, Germany), filtered with a 900SP laser blocker filter, split with a 560lp dichroic mirror, bandpass filtered with an ET620/60 (Chroma Technology GmbH, Germany), and detected by a GaAsP PMT detector (H7422A‐40, Hamamatsu, Japan). Samples were imaged with a 0.70 µm pixel size and 2‐line averaging. Z‐stacks were performed with a 1 µm step size.

#### 5.5.3 Transmission Electron Microscopy (TEM)

MEF PCL scaffolds with ACPCs chondrogenically differentiated for 28 days were fixed in 4% paraformaldehyde, rinsed, and stored in PBS at 4 degrees until imaging. Samples were cut transversely into parallelepipeds and re‐fixed with 2.5% paraformaldehyde (PFA), 2% glutaraldehyde, 0.18 M sucrose, and 0.1% picric acid for 12 h. The samples were then post‐fixed with osmium tetroxide (OsO4), 1% twice, with intermediate steps using thiocarbohydrazide (TCH), lead aspartate, and uranyl acetate for enhanced contrast. Samples were dehydrated in acetone baths of increasing concentrations, followed by gradual infiltration of EPON resin/acetone mixes. Samples were cured in pure EPON resin for 3 days before sectioning transversely to the main axis. 70 nm ultrathin sections were cut (Ultracut Ultramicrotome, Leica Microsystems), collected on formvar and carbon‐coated TEM grids, and stained with uranyl acetate and lead citrate (Leica AC20). Micrographs were collected on a Tecnai T12 (FEI Thermo Fisher, Lab6 filament) operating at 80 kV, equipped with a Veleta 2k × 2k CCD camera (EMSIS, Munster, Germany). Collagen fibrils in TEM images were segmented by thresholding, and individual particles were analyzed in Fiji. Aspect ratio (AR) measurements were extracted from the resulting particle analysis. To quantify fibril orientation, a custom Fiji macro was used to color‐code fibrils according to AR: cyan for 1 ≤ AR ≤ 2, gray for 2 < AR ≤ 3, and blue for AR > 3. This approach allowed mapping of AR values onto individual fibrils for quantitative and qualitative assessment of collagen directionality. Collagen fibril diameter and D‐banding periodicity were measured on ten individual fibrils using ImageJ2 (Version 2.14.0), with diameters obtained from particle analysis and D‐banding determined via fast Fourier transform (FFT).

### 5.6 Statistics

All statistical analyses were performed in GraphPad Prism 11.0.0. Comparisons between two groups were made using an unpaired *t*‐test assuming Gaussian‐distributed data. Comparisons among more than two groups were performed using one‐way ANOVA with Tukey's multiple comparisons test. Two‐way ANOVAs were performed with *α* = 0.05 and Tukey's multiple comparisons test. Differences were considered statistically significant at *p* < 0.05. Data are presented as mean ± standard deviation.

For fabrication and structural measurements (Figures 1 and 2), n represents the number of independent prints, fibers, or fibrils measured, with multiple regions assessed per construct where applicable. For mechanical testing (Figure 3) and biological analyses (Figures 4, 5, 6), *n* represents the number of independent scaffolds or constructs analyzed per condition. ACPCs were obtained from a single donor; all other replicates are technical. The exact *n* for each analysis is stated in the corresponding figure legend. For qualitative imaging data, representative images are shown with sample and field numbers specified in the figure legend.

## Author Contributions

A.P.M. and L.S. contributed to the conceptualization, experimental design, execution of experiments, data analysis, and manuscript writing. J.L.M.R. assisted with the experimental execution and data analysis on material chemistry and was involved in writing the manuscript. A.P.M. and J.L.M.R. revised the manuscript. A.G.G. assisted with the experimental execution of MEW and MEF fabrication. I.M. assisted with the experimental execution and data analysis regarding collagen imaging. S.G. and A.K.O. contributed to the analytical procedures regarding cell behavior. M.R. and J.G. provided foundational insights that guided the study. M.d.R. contributed to study conceptualization. J.M. was involved in conceptualization, overall supervision, and contributed to data interpretation and writing the manuscript.

## Funding

This publication is part of the project the Dutch Research Council Gravitation Program “Materials Driven Regeneration” (024.003.013), LS‐CarE (with project number NWA.1389.20.192) of the research program NWA‐ORC which is (partly) financed by the Dutch Research Council (NWO), the Dutch Research Council Summit Program “DRIVE‐RM” (SUMMIT.1.027) and the Horizon Europe ERC‐advanced “Re‐COLL” (101142963),

## Conflicts of Interest

The authors declare no conflicts of interest.

## Ethics Statement

Procedures regarding cell harvesting and use were followed according to the guidelines of the Ethical and Animal Welfare body of Utrecht University.

## Supporting information




**Supporting File**: adma75138‐sup‐0001‐SuppMat.pdf.

## Data Availability

The data that support the findings of this study are available from the corresponding author upon reasonable request.
