# Supplementary material for: Fabrication of Aligned Polycaprolactone Nanofibrillar Bundles to Control Cell‐Mediated Collagen Deposition in Engineered Cartilage
Source: Adv Mater. 2026 Sep 24;38(62):e75138. doi: 10.1002/adma.75138 (PMC7619664; doi:10.1002/adma.75138)
Supplement: Supplementary file 1 [file ADMA-38-e75138-s001.pdf]

Supporting information

Fabrication of aligned polycaprolactone nanofibrillar bundles to control cell-mediated collagen deposition in engineered cartilage

Alba Pueyo Moliner[1,2#], Lennard Spauwen[1,3#], James L. Martin Robinson[1], Ángela Garcia Grech[1], Isabelle Martinier[1], Sara Grasselli[1], Aylin Kara Özenler[1], Matthias Ryma[4], Jürgen Groll[4], Mylène de Ruijter[1,3], Jos Malda[1,3]*

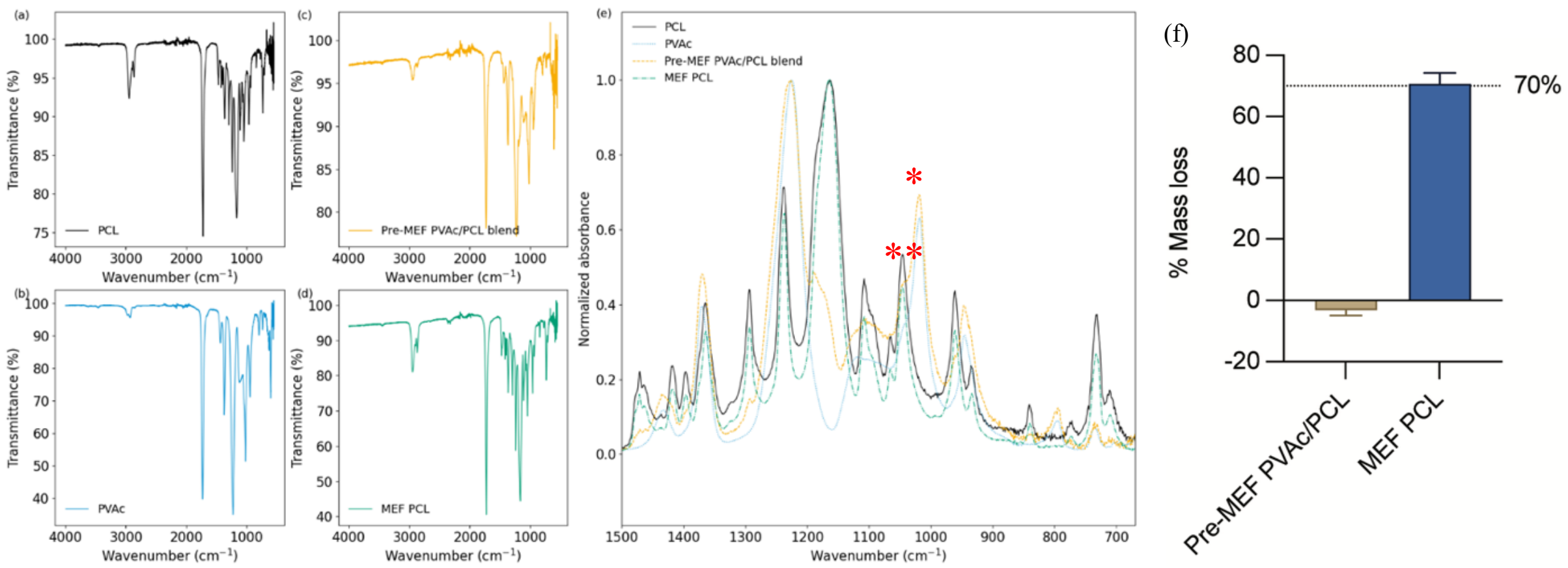

Figure S1. *Fourier transform infrared spectroscopy of pre-fibrillated (pre-MEF PVAc-PCL blend) and fibrillated (MEF PCL) mixtures of MEW printed PVAc and PCL. (a) Virgin PCL pellets. (b) Virgin PVAc pellets. (c) pre-MEF PVAc-PCL blend. (d) MEF PCL. (e) Overlap of the normalized spectra of all the samples. (f) Mass loss following treatment of pre-MEF PVAc/PCL with ethanol for PVAc removal (right, blue) and PBS as a control (left, brown), n=16 independent scaffolds. Percentage mass loss was calculated relative to the initial scaffold mass.*

The melt electrofibrillation (MEF) procedure involves the selective dissolution of the polyvinyl acetate (PVAc) component of a melt electrowritten blend of PVAc and polycaprolactone (PCL). We used Fourier transform infrared (FTIR) spectroscopy to follow this selective dissolution. The spectra of the printed pre-MEF PVAc-PCL blend is shown in Figure 1(c), which is a combination of the spectra of the virgin non-printed PCL and PVAc precursors which form the blend, shown in part (a) and (b) respectively. Figure 1(d) shows the spectra for the fibrillated polymer blend (MEF PCL), where the PVAc component has been selectively dissolved using ethanol. Figure 1(e) shows the overlapped absorption spectra for all samples. The individual attenuated total reflectance (ATR) spectra of each sample were normalized to allow for a qualitative comparison between them. The spectra for virgin-PCL and MEW-PCL were normalized to the peak at 1163 cm^{-1}, and the PVAc and pre-MEF samples were normalized to the most intense peaks in their spectra (~1226 cm^{-1}). The overlapped spectra qualitatively indicates that the pre-MEF sample indeed contains both PVAc and PCL, as can be seen from the presence of the * (~1019 cm^{-1}) peak in PVAc and the pre-MEF PVAc/PCL samples as well as the shoulder peak ** (~1163 cm^{-1}) which results from the presence of PCL. In contrast to this, the

fibrillated sample, MEF PCL, shows no * peak and generally matches the spectrum of the virgin-PCL very closely. These results qualitatively indicate the full removal of the PVAc from the fibrillated printed blends. In addition, pre-MEF PVAc/PCL blend contained 70 wt% PVAc and 30 wt% PCL, and the measured mass loss following fibrillation was consistent with the expected removal of the sacrificial PVAc phase. MEF PCL constructs were treated with PBS as controls.

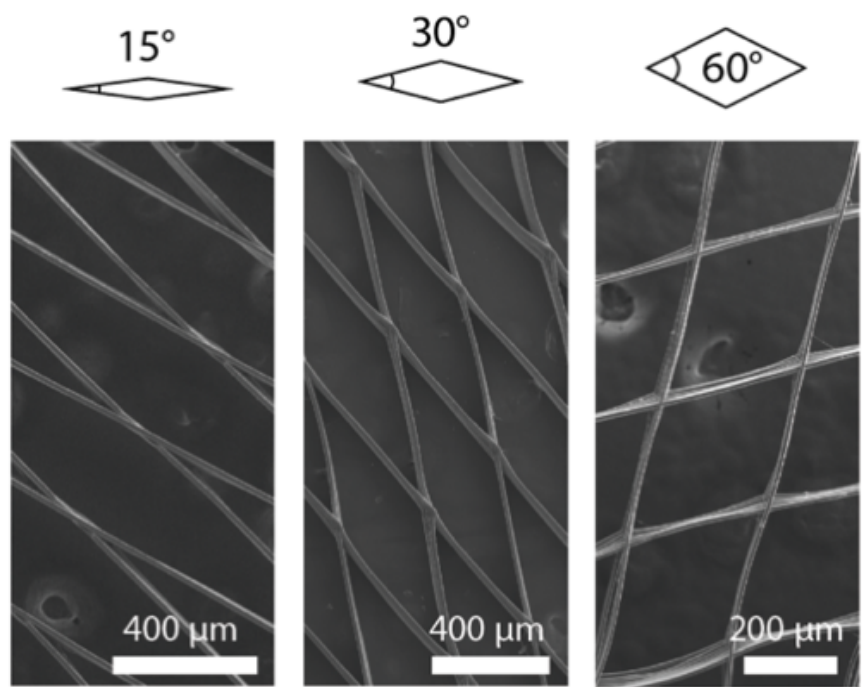

Figure S2. *SEM images of rhombus MEW PCL scaffolds with angles of 15°, 30° and 60° (25 layers).*

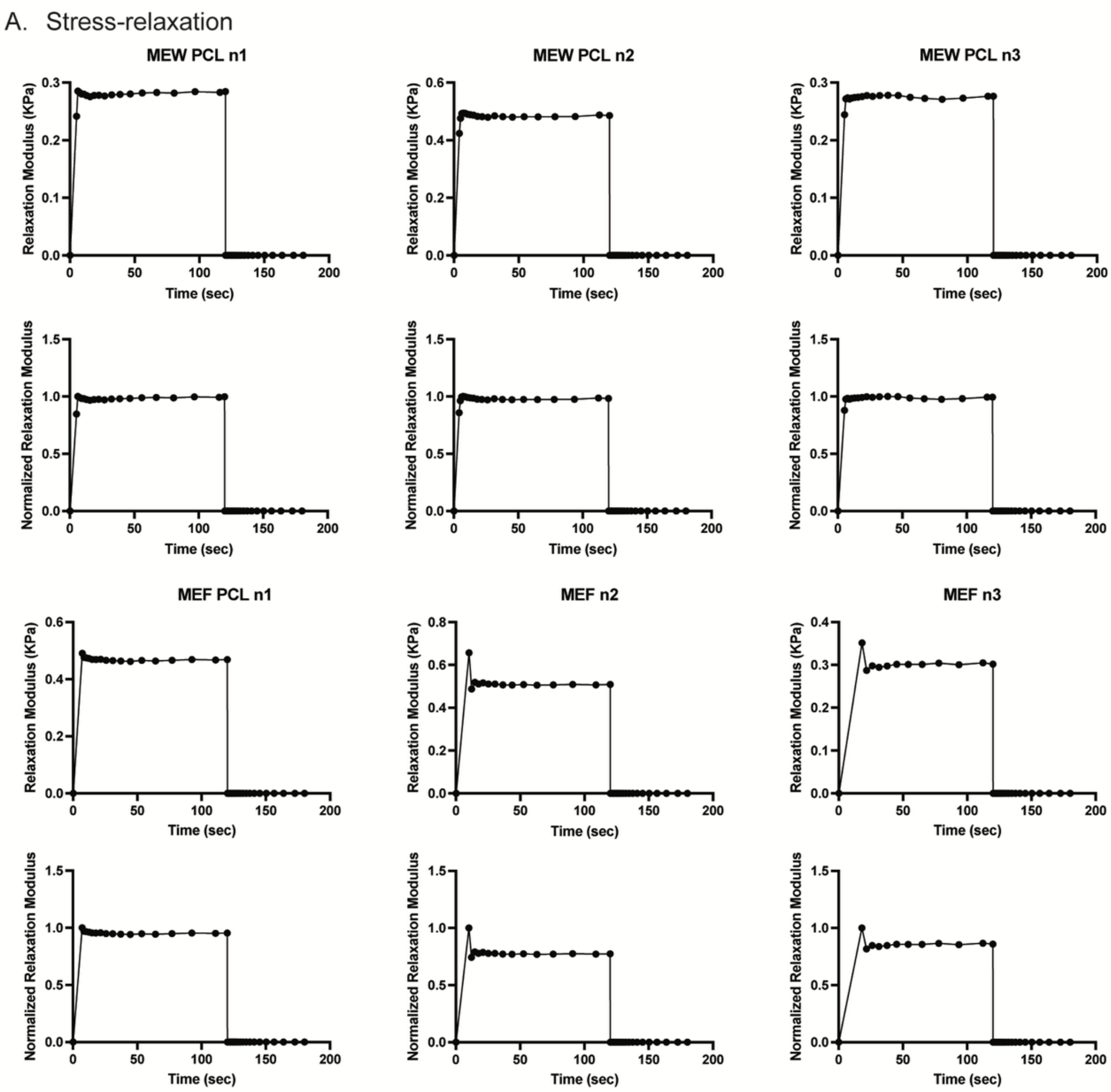

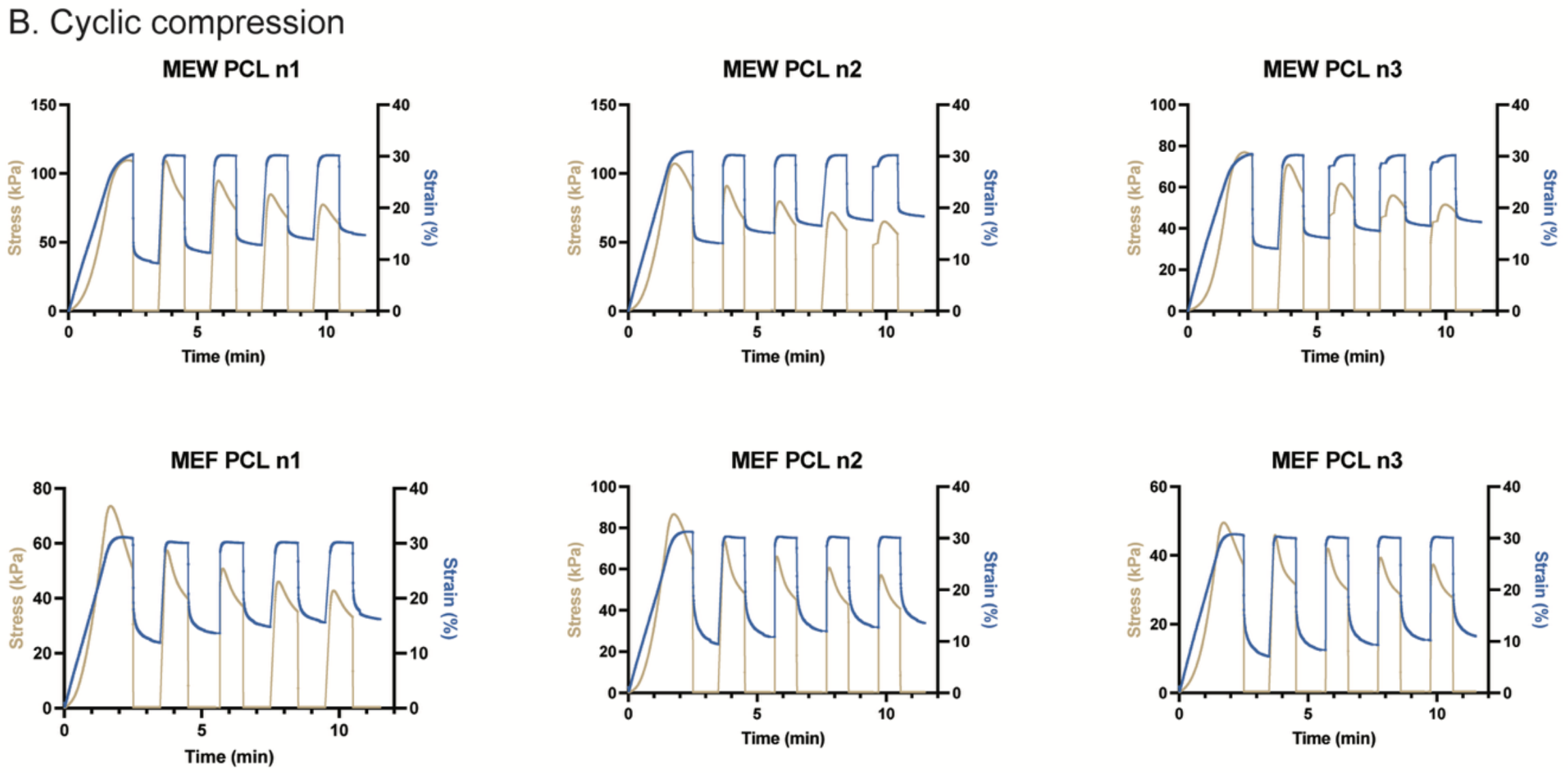

Figure S3. *(a) Stress relaxation behavior of MEW and MEF PCL scaffolds. Stress relaxation responses of n=3 independent MEW scaffold samples (top) and n=3 independent MEF scaffold samples (bottom). For each scaffold, relaxation modulus (kPa) is plotted against time (s), while the corresponding normalized relaxation modulus is shown in the lower graphs. (b) Cyclic compression profiles of MEW and MEF PCL scaffolds. Representative stress, strain, and time profiles recorded during cyclic compression testing of n=3 independent MEW scaffold samples (top) and three MEF scaffold samples (bottom). Stress (kPa) is shown on the left y-axis, strain (%) on the right y-axis, and time (min) on the x-axis.*

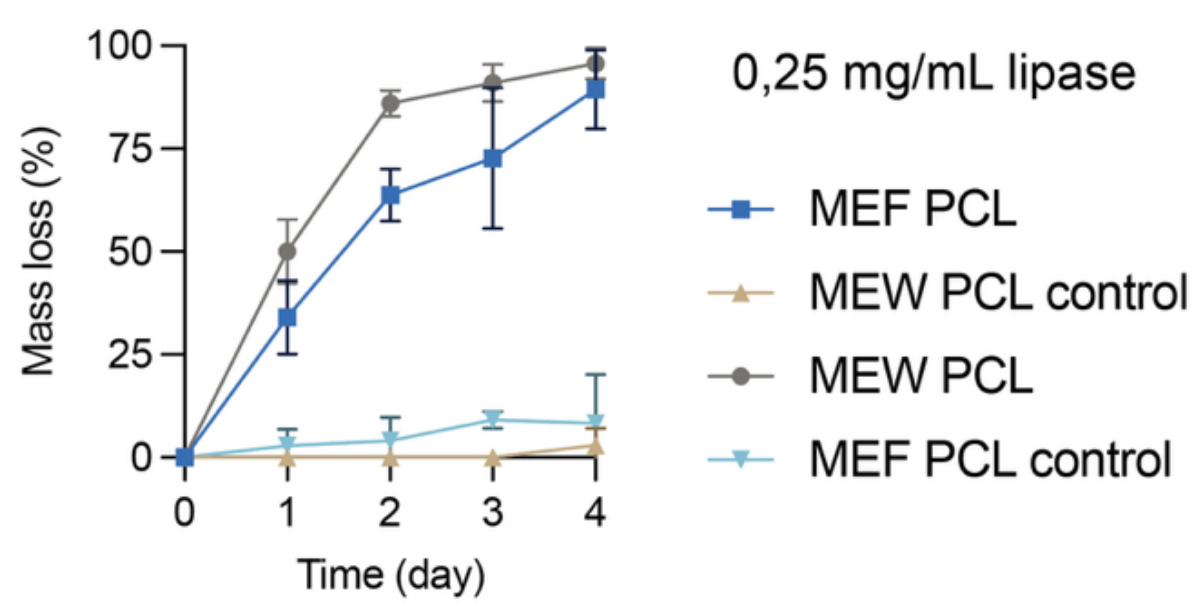

Figure S4. *Enzymatic degradation of MEW and MEF PCL scaffolds. Percentage mass loss of MEW and MEF PCL scaffolds following incubation with lipase for up to 4 days. Lipase-treated MEF and MEW scaffolds exhibited increasing mass loss over time, whereas the corresponding PBS controls showed negligible mass change throughout the experiment. Data are presented as mean ± SD.*

PCL is a slowly degrading, bulk-eroding polyester whose *in vivo* degradation is dominated by hydrolytic cleavage of ester bonds. Degradation typically proceeds in two stages: first, random chain scission

lowers molecular weight with little immediate mass loss or visible deformation; second, once a critical molecular-weight threshold is reached, the material begins to lose mass and structural integrity. Long-term *in vivo* studies indicate that PCL can remain structurally intact for extended periods, with substantial degradation occurring over years rather than weeks or months. Accordingly, our lipase-based assay is best viewed as an accelerated in vitro approximation of the long-term degradation process rather than a direct mimic of the in vivo setting. This distinction is relevant because scaffold persistence is expected to support maintenance of the newly deposited collagen type II network by preserving the topographic cues that guide matrix organization, whereas progressive scaffold degradation may allow tissue remodelling and potentially reduce alignment stability.

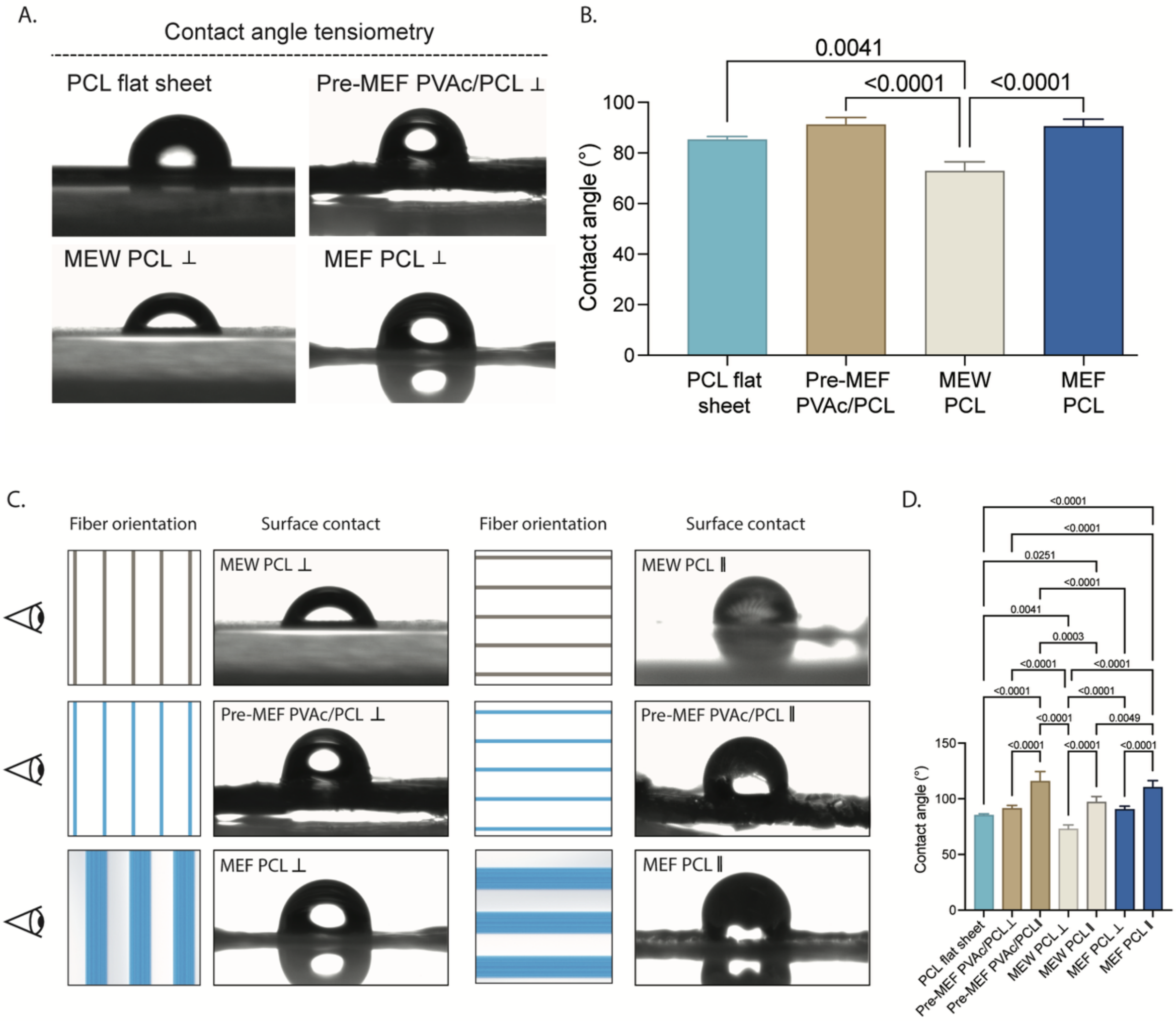

Figure S5. *Contact angle measurements of MEW printed neat PCL (MEW-PCL), pre-fibrillated PVAc-PCL blend (pre-MEF PVAc-PCL blend) and fibrillated PCL (MEF PCL) samples. (a) Representative images of sessile drop measurements used for contact angle analysis. (b) Quantification of contact angles measured perpendicular to the fiber direction for Pre-MEF PVAc/PCL (n = 4), MEW PCL (n = 5), and MEF PCL (n = 9), including a flat PCL sheet as a control (n = 4). (c) Diagram of the orientation of the fibres with respect to the image capture and representative images of the sessile drops. (d)*

Supporting information

Summary of the different contact angles measured in a perpendicular and parallel orientation of the different samples, including measurements on a flat PCL sheet for comparison.

To assess the differences in wettability between the different scaffold types used, we performed sessile drop contact angle measurements. The samples used in the main text present anisotropic, fibrous, and porous structures composed of box-shaped scaffolds. The arrangement of these fibres, as well as the chemistry of the materials that compose them, will influence the macroscopic wetting properties of the sessile drops laid onto them. To attempt to make the measurements more representative and relevant to the environment that cells will experience in the scaffolds, measurements with drops placed directly onto the top of the scaffolds were not carried out. Instead, we aimed to mimic the structure of fibres in the walls of the box scaffolds by measuring fibres printed in a singular direction and laid flat with respect to the drop being measured.

The MEW PCL samples were composed of stacked, fully fused, printed MEW fibres laid flat. The pre-MEF PVAc/PCL measurements samples were composed of close packed fibres which were directly printed flat. These samples were then fibrillated to create the MEF PCL samples which were composed of similarly close, now fibrillated, bundles of PCL fibres. The packing density of the pre-MEF and MEF samples is similar to what can be seen from the walls of their corresponding box-shaped scaffolds (Figure S3, A,B) and represent a qualitative approximation to them.

Due to the directional nature of the measurement samples, a difference in the wettability along and across the fibres might be expected and therefore measurements in these two different orientations were carried out. Considering that FTIR data indicates that the post-fibrillation MEF PCL samples do not contain any PVAc, differences in wettability between flat PCL sheets, MEW PCL and MEF PCL samples will be due to differences in the topography of the samples. In the case of pre-MEF PVAc/PCL blend samples the presence of the PVAc is expect to also modify the wettability.

The results for this analysis are shown in Figure 4D. There is a clear trend in the wettability of each sample along (perpendicular view) and across the fibres (parallel view). For all samples higher contact angles were seen for the samples in the parallel view compared to the perpendicular view. This is a result of contact line pinning due to periodic increases in height across the fibre directions. See, for example, this recent paper discussing this topic further: Soft Matter, 2007,3, 1163-1169. Comparing the wettability of MEW PCL samples, which are composed of fully fused parallel-aligned fibres, to that of the flat PCL sheets we observe that the contact angle is higher for the MEW-PCL in the parallel direction and lower in the perpendicular direction. Following from the reference mentioned above, this is to be expected due to the contact line pinning across the fibres and a surface area enhanced wettability along the fibres (Wenzel wetting effect).

Supporting information

Between the different printed samples, we see the same trend in the wettability both in the perpendicular and parallel directions. MEW PCL shows the lowest contact angle which then increase for MEF PCL and, then further, for pre-MEF PVAc/PCL samples. The horizontal parallel printing of the pre-MEF PVAc/PCL fibres leads to a much rougher (height differences between adjacent fibres) and less ordered structure than what can be achieved from the direct vertical stacking of MEW PCL fibres. The resulting post-fibrillation samples, MEF PCL, inherit this less ordered structure. Differences in wettability between MEW PCL and MEF PCL samples, which are chemically identical, can be attributed to this difference between the samples. Along the fibres, we might expect a decrease in the contact angle, as per the Wenzel wetting behaviour discussed above. However, due to spaces between the fibres, the air gaps present in the sample will counteract the increased surface area of the fibres themselves. For the MEF PCL samples we expect, at least right as the drop is deposited and measured, for the wetting to fall withing a Cassie-Baxter regime. See, for example, Langmuir 2014, 30, 2061−2067 for a description of this regime. The presence of air gaps in the material will increase the contact angle. Across the fibres (in the parallel view) this change in wetting regime and, importantly, much higher roughness also leads to an increase in the contact angle (parallel view) with respect to MEW PCL.

The difference in contact angles between pre and post MEF samples is not that significant which potentially results from their similar topography. It seems that the present of PVAc in the pre-MEF PVAc/PCL samples does not alter the wetting behaviour substantially.

It is worth considering the relevance of these measurements to the environment experienced by cells within the constructs. The contact-angle measurements were performed under dry conditions, and the presence of air gaps between the MEF PCL fibres during the sessile-drop measurement differs from the fully hydrated, GelMA- and medium-filled environment of the scaffolds during cell culture. Therefore, these measurements should be interpreted primarily as a comparative assessment of dry-state surface wetting rather than a direct representation of the cellular microenvironment. Nevertheless, the relatively small differences in dry-state wettability between MEW PCL and MEF PCL suggest that differences in surface wettability alone are unlikely to fully account for the distinct cellular responses observed, although a contribution of wettability under hydrated conditions cannot be excluded. Other surface and topographical cues may therefore also contribute to the observed cellular behaviour.

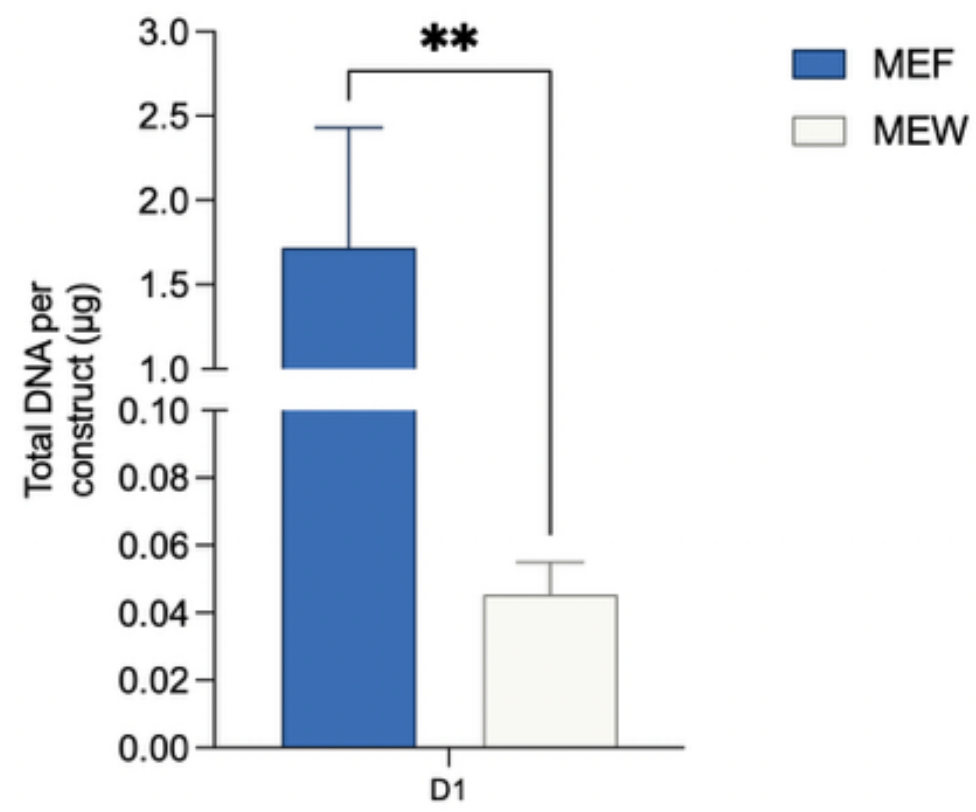

Figure S6. *Initial cell attachment to MEF and MEW PCL scaffolds. Total DNA content per construct was quantified at Day 1 using a PicoGreen assay as a measure of initial cell attachment and retention. MEF PCL scaffolds exhibited higher DNA content compared with MEW PCL scaffolds, indicating greater initial cell attachment. (n = 6 independent scaffolds per condition).*

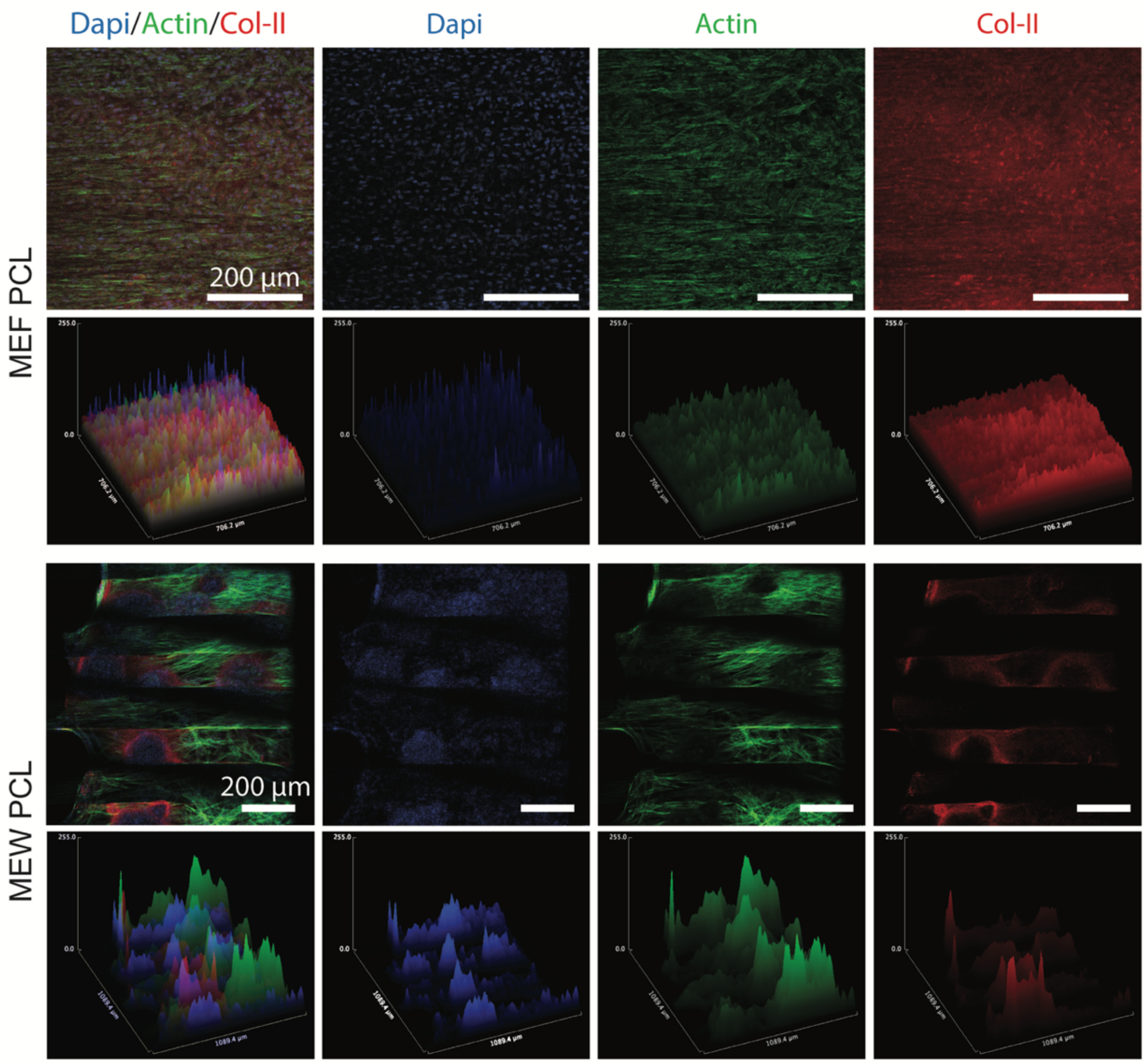

Figure S7. *Representative fluorescence microscopy images showing individual channels corresponding to the merged images presented in Figure 4E. Nuclei are shown in blue (DAPI), actin cytoskeleton in green, and collagen type II in red.*

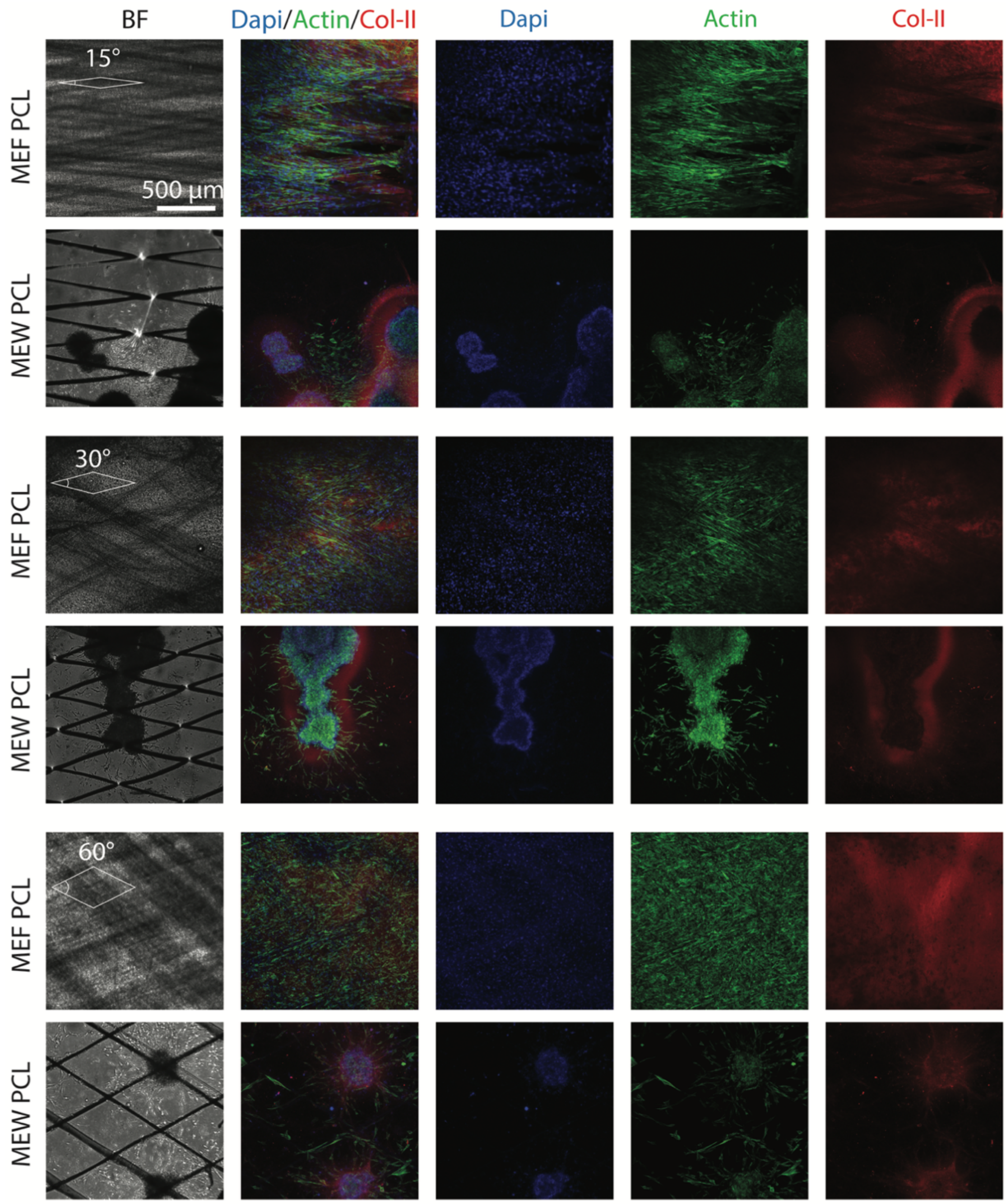

Figure S8. *Representative brightfield and fluorescence microscopy images showing individual channels corresponding to the merged images presented in Figure 4G, of ACPC-laden rhombus-shaped scaffolds with angles ranging from 15° to 60° (scale bar: 500 µm). Nuclei are shown in blue (DAPI), actin cytoskeleton in green, and collagen type II in red.*

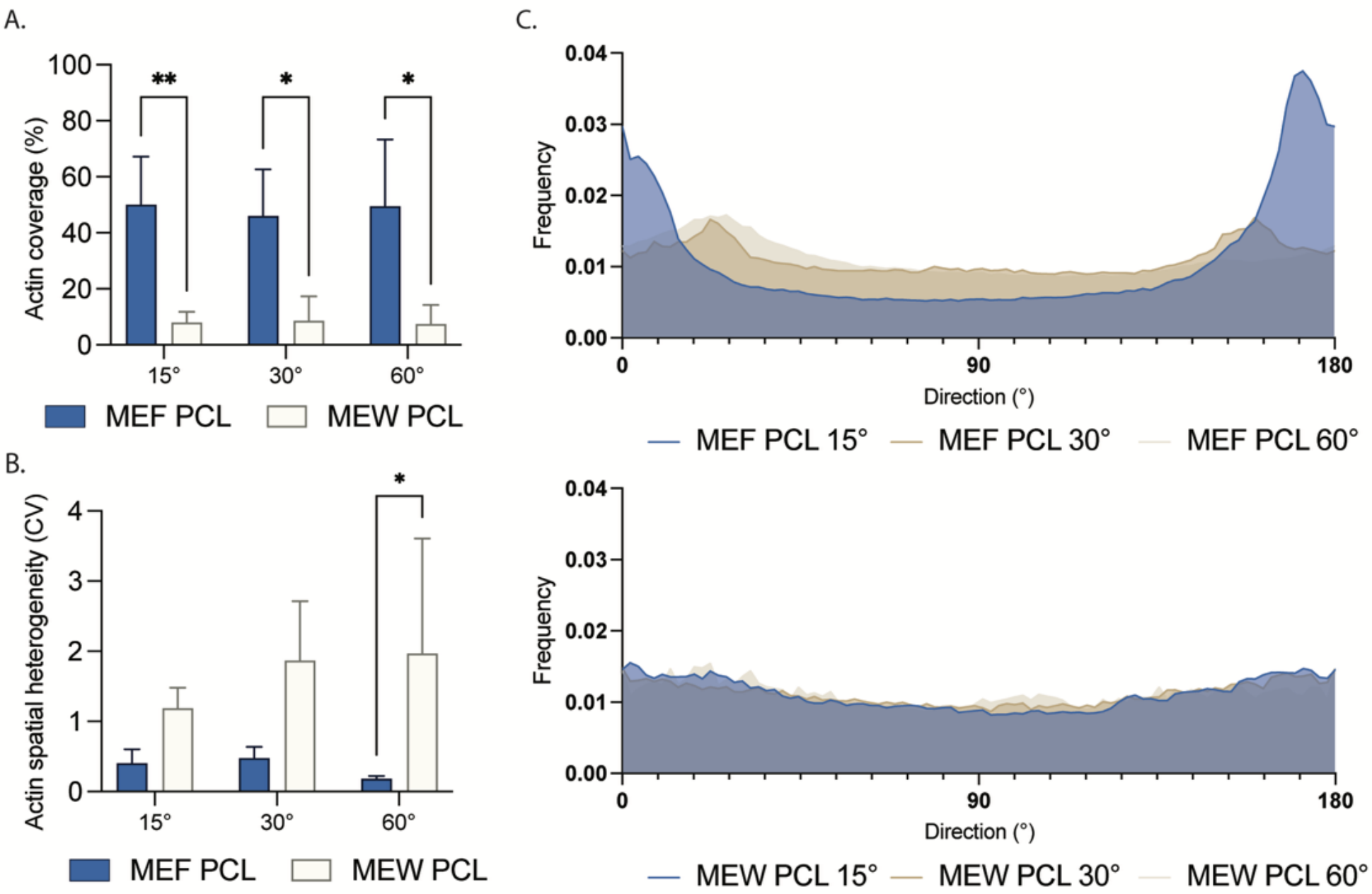

Figure S9. *Quantitative analysis of cellular coverage, spatial heterogeneity, and actin orientation on MEW and MEF PCL scaffolds. (a) Quantification of F-actin-positive area (%) for MEW and MEF PCL scaffolds with rhombus patterns printed at 15°, 30°, and 60° (n=3). (b) Quantification of the spatial heterogeneity of F-actin distribution, expressed as the coefficient of variation (CV) of F-actin-positive area measured across 25 equal regions within each image, for MEW and MEF PCL scaffolds at 15°, 30°, and 60° (n=3). Higher CV values indicate greater spatial heterogeneity of the cellular distribution. (c) Orientation distribution of F-actin for MEF and MEW PCL scaffolds at 15°, 30°, and 60°, shown as frequency as a function of orientation angle. MEF scaffolds exhibit pronounced orientation peaks, corresponding to the predominant orientation of the underlying rhombus PCL fibers, whereas MEW scaffolds show a less pronounced directional distribution.*

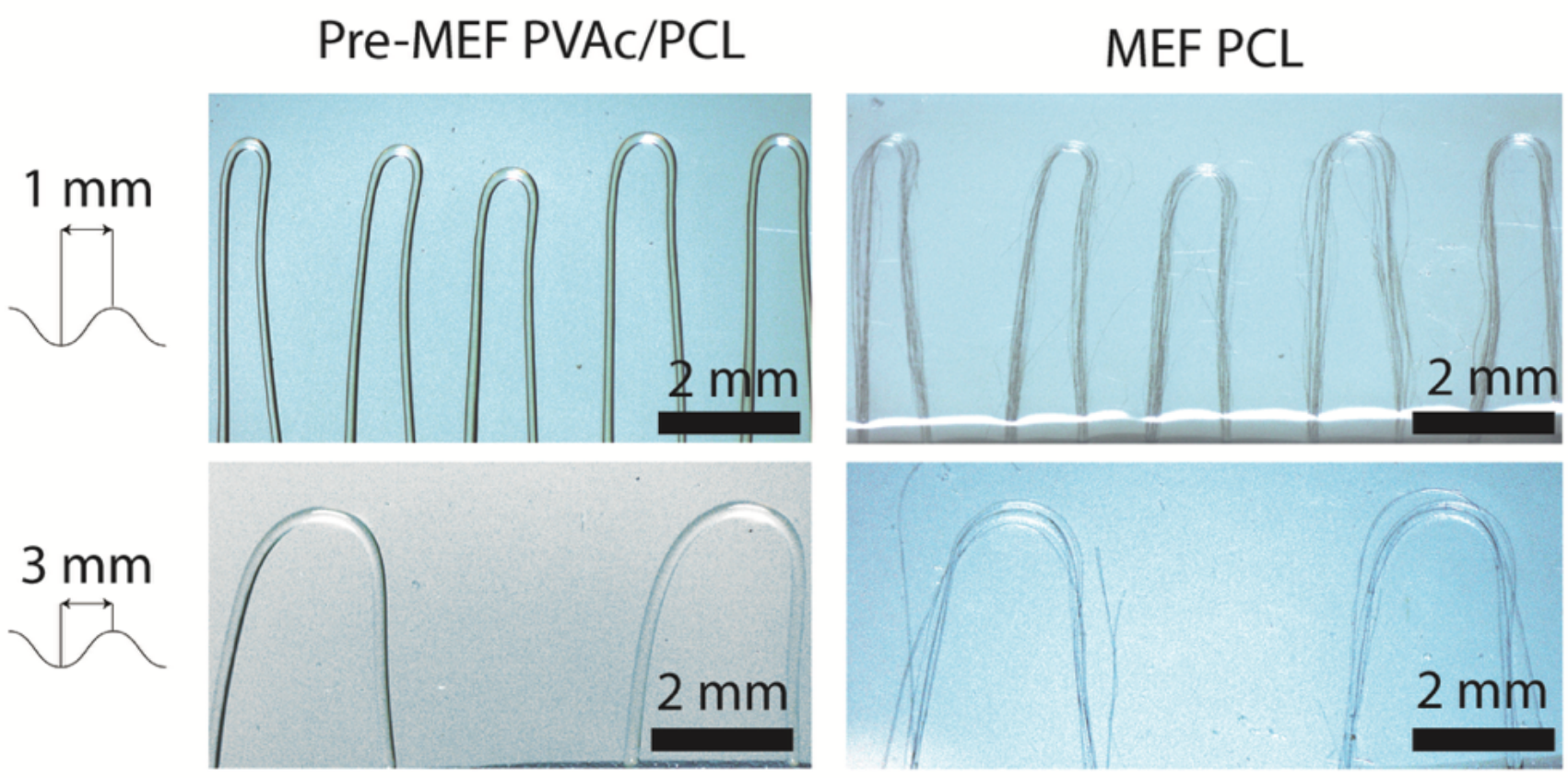

Figure S10. *Pre-MEF PVAc/PCL printed a single sinusoidal wave function with a wavelength of 1 and 3 mm, imaged with brightfield pre and post fibrillation into MEF PCL*

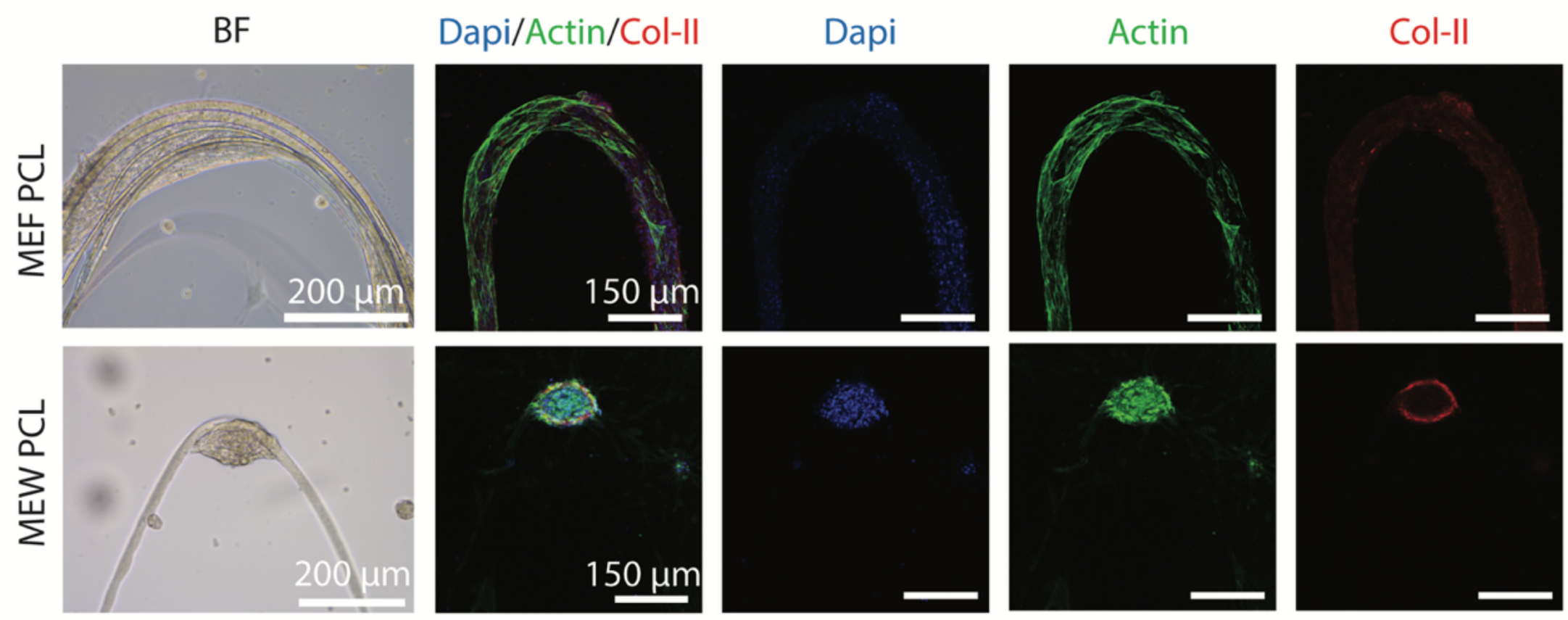

Figure S11. *Representative fluorescence microscopy images showing individual channels corresponding to the merged images presented in Figure 4H, of cell-laden MEF PCL and MEW PCL curved scaffolds. Nuclei are shown in blue (DAPI), actin in green, and collagen type II in red.*

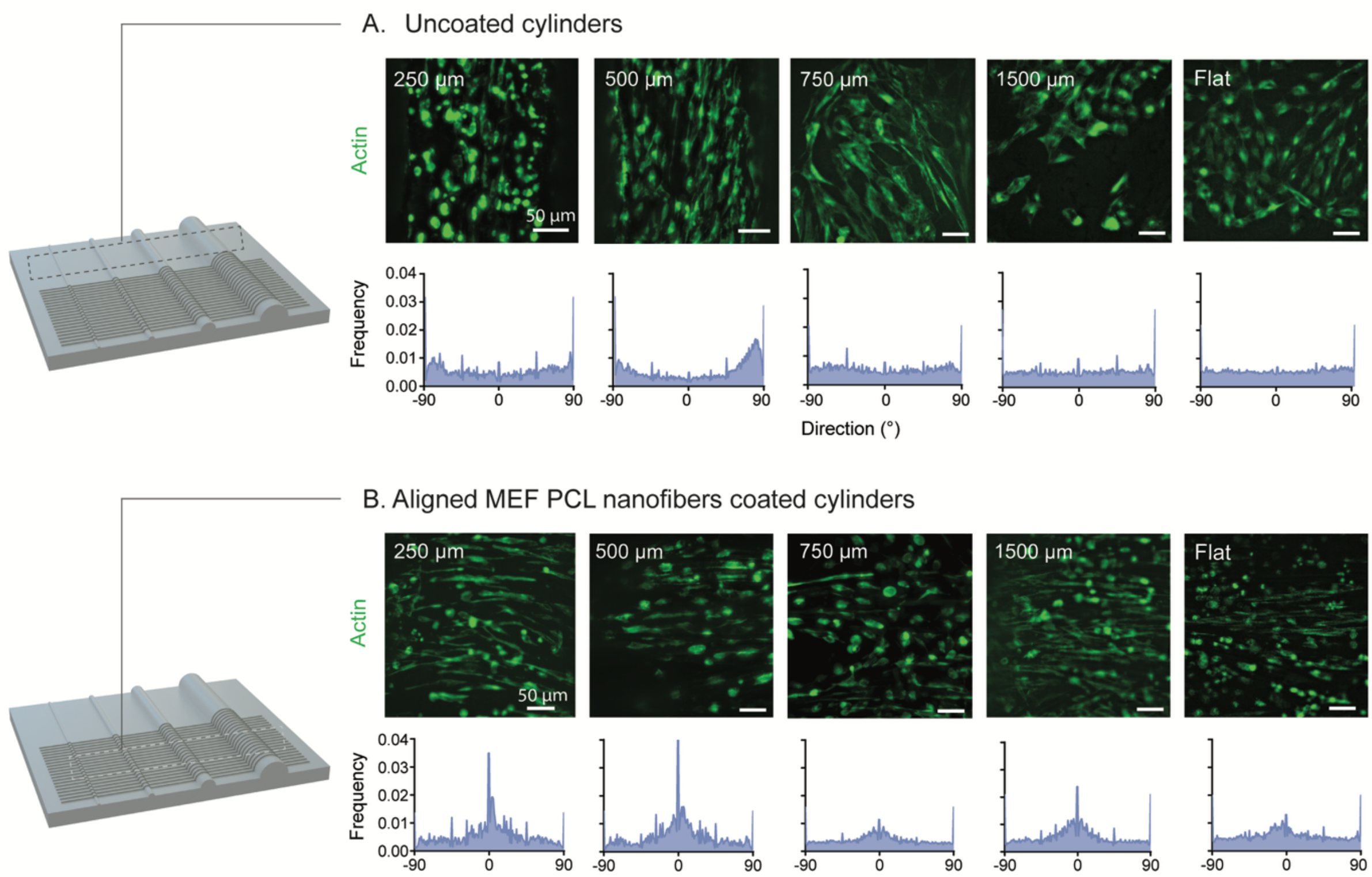

Figure S12. *Contact guidance of ACPCs on MEF PCL nanofiber bundles on curved substrates. Half-cylindrical substrates with defined diameters (250–1500 µm) were used to investigate the interplay between mesoscale curvature and MEF-derived topographical cues. Surfaces were either A) uncoated or B) overlaid with aligned MEF PCL fiber bundles.*

On uncoated controls, ACPCs aligned along the cylinders' axes only at small diameters (≤500 µm), consistent with curvature-driven alignment. In contrast, on identical cylinder substrates coated in MEF fibres, ACPCs consistently aligned along the fiber direction regardless of cylinder diameter or underlying curvature, indicating that contact guidance from MEF topography dominates over curvature effects. The hierarchical nanofibrillar architecture of MEF, with subcellular fiber dimensions and ECM-like spacing, provides strong guidance cues that promote robust cell alignment.

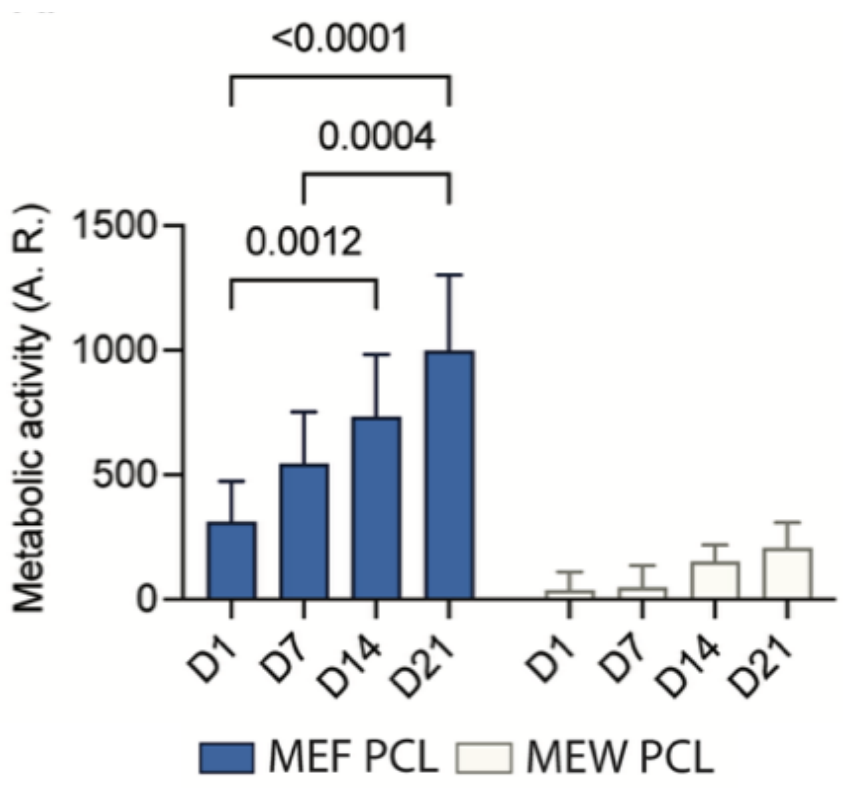

Figure S13. *Metabolic activity of ACPCs cultured on MEF and MEW PCL scaffolds chondrogenically differentiated over a 21-day period.*

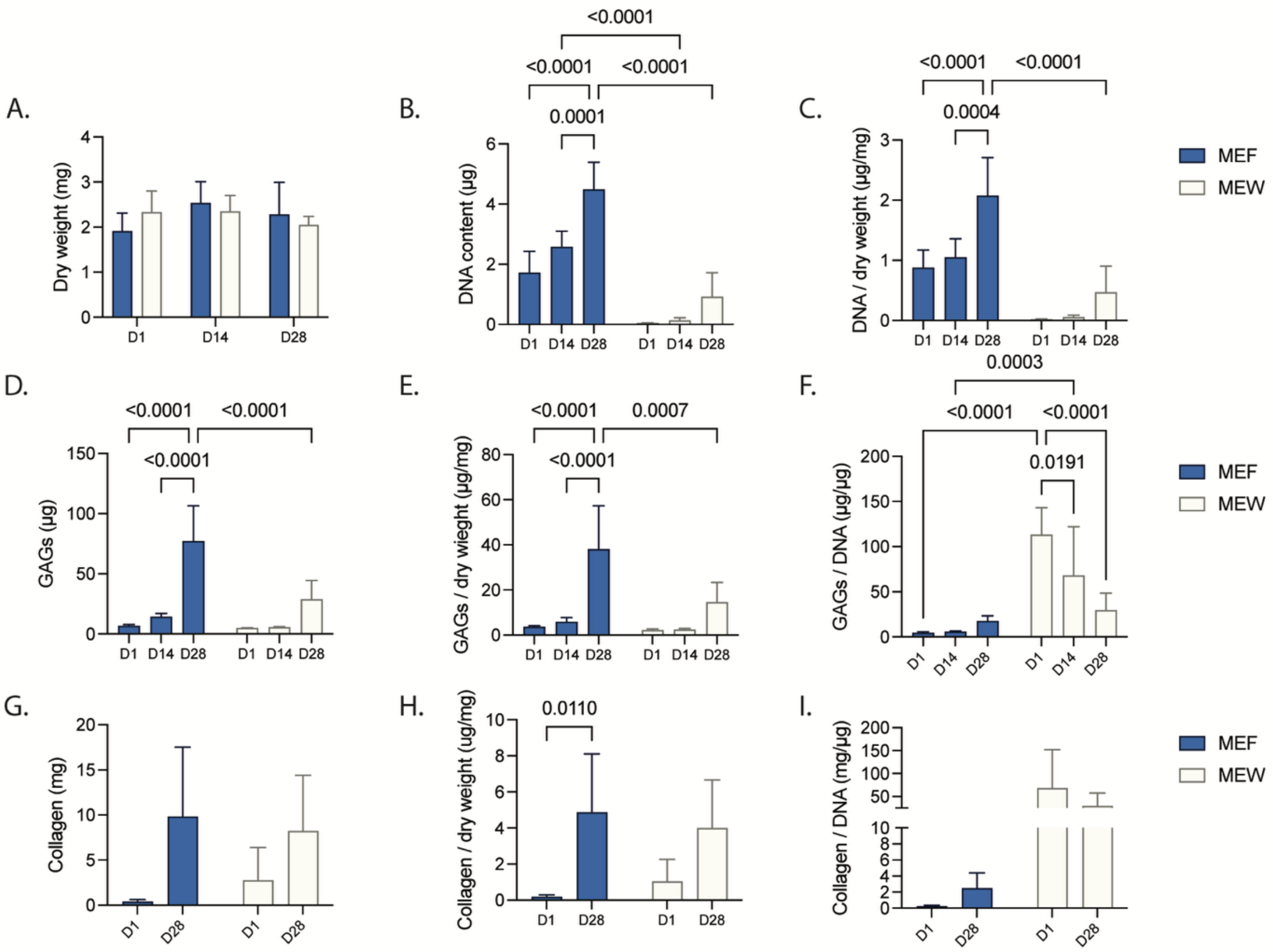

Figure S14. *Quantitative biochemical analysis of extracellular matrix deposition by ACPCs cultured on MEF and MEW PCL scaffolds under chondrogenic conditions.(a) Dry weight (mg), (b) DNA content (µg), (c) DNA content normalized to dry weight (µg/mg),(d) glycosaminoglycan (GAG) content (µg),(e) GAG content normalized to dry weight (µg/mg), (f) GAG content normalized to DNA (µg/µg), (g) total collagen content (mg), (h) total collagen content normalized to dry weight (mg/mg) and (i) total collagen content normalized to DNA content (I, mg/µg) were quantified after 1, 14, and 28 days of culture. GAG content was determined using the DMMB assay, total collagen content using the hydroxyproline assay*

and DNA using the picogreen assay. Data are presented as mean ± SD from one donor with six independent scaffolds (n = 6).

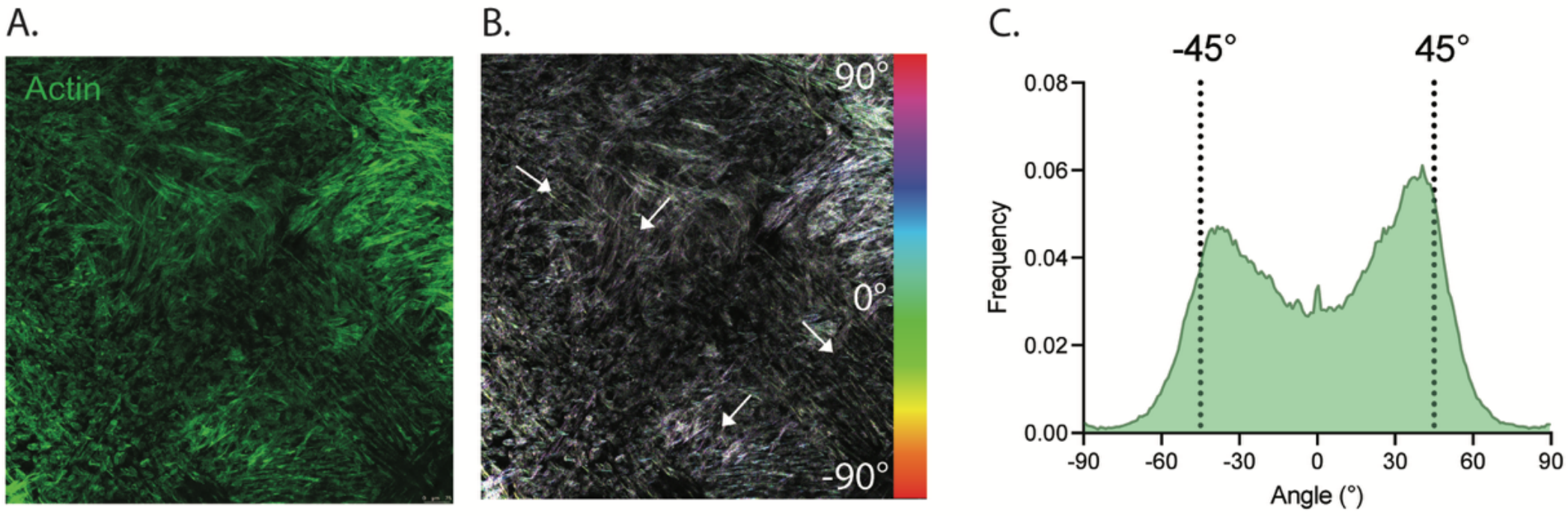

Figure S15. *F-actin orientation of cells cultured on the thick sinusoidal MEF scaffold. (a) Representative fluorescence image showing F-actin organization of cells cultured on the thick sinusoidal/arched MEF construct presented in Figure 6. (b) Corresponding orientation colour map illustrating the local orientation of the F-actin cytoskeleton. (c) Frequency distribution of F-actin orientations, showing the preferential orientation of cellular F-actin along the underlying scaffold architecture. Orientation angles range from −45° to 45°.*
